# The injection-production performance of an enhanced geothermal system considering fracture network complexity and thermo-hydro-mechanical coupling in numerical simulations

**DOI:** 10.1038/s41598-023-41745-7

**Published:** 2023-09-04

**Authors:** Zhihong Lei, Yulong Zhang, Qiliang Cui, Yu Shi

**Affiliations:** Faculty of Geosciences and Environmental Engineering, Southwest Jiaotong University, Chengdu, 611756 China

**Keywords:** Geothermal energy, Software

## Abstract

The effect of fracture networks on EGS performance remains worth further investigation to guide the formulation of geothermal extraction strategy. We established models that account for thermo-hydraulic-mechanical (THM) coupling and that are based on the framework of discrete fracture network (DFN) to evaluate the heat extraction performance in deep-seated fractured reservoir. Our numerical results reveal that the zones of temperature, pressure, and stress perturbation diffuse asynchronously during the circulation of injection-production, and the stress perturbation always lags behind the other two. Furthermore, the effects of the fracture network characteristics including randomness, geometry, length, aperture, and injection parameters on the heat production are quantitatively investigated. Under the same number of fractures, different network geometry leads to different EGS production performance, the network with horizontal fracture set shows better thermal extraction performance but poor injection performance, which is because the fracture dip affects the thermal evolution on the horizontal plane. The effect of fracture length on EGS performance highly depends on its orientation, the excessive increase of fracture length towards injection-production wells is detrimental to heat extraction. The fracture aperture affects the working fluid transport and thus the EGS performance, the fractured reservoir with smaller fracture aperture shows the worse fluid flow performance but the better geothermal extraction performance, thus we believe that the optimal fracture aperture should be kept at a level of 0.5–1.0 mm in a self-propping fractured granitic system. The main influence of injection parameters on thermal extraction from the fractured reservoirs is the injection mass rate, because a high injection rate results in significant solid responses, including failure stress concentration, decreased safety factor, and increased permeability, which occur in those fractures that are originally connected to the injection well. These results of our research and the insights obtained have important implications for deep geothermal geoengineering activities.

## Introduction

Traditional fossil fuels are finite resources and will be eventually depleted due to rapid growth in world population. Developing geothermal energy is an effective strategy in the alleviating of current energy crisis^1–3^. As an alternative energy, geothermal energy is clean, renewable and most promising^1, 4^. Hot dry rock (HDR) is deep-seated geothermal resources, which refers to high-temperature rocks with extremely low permeability^5, 6^. HDR resources can be exploited by injecting low-temperature working fluid to carry heat to the wellhead for power generation and heating, and thus the key of developing a HDR engineering is to improve the permeability of the impermeable reservoir.

**Figure 1:**
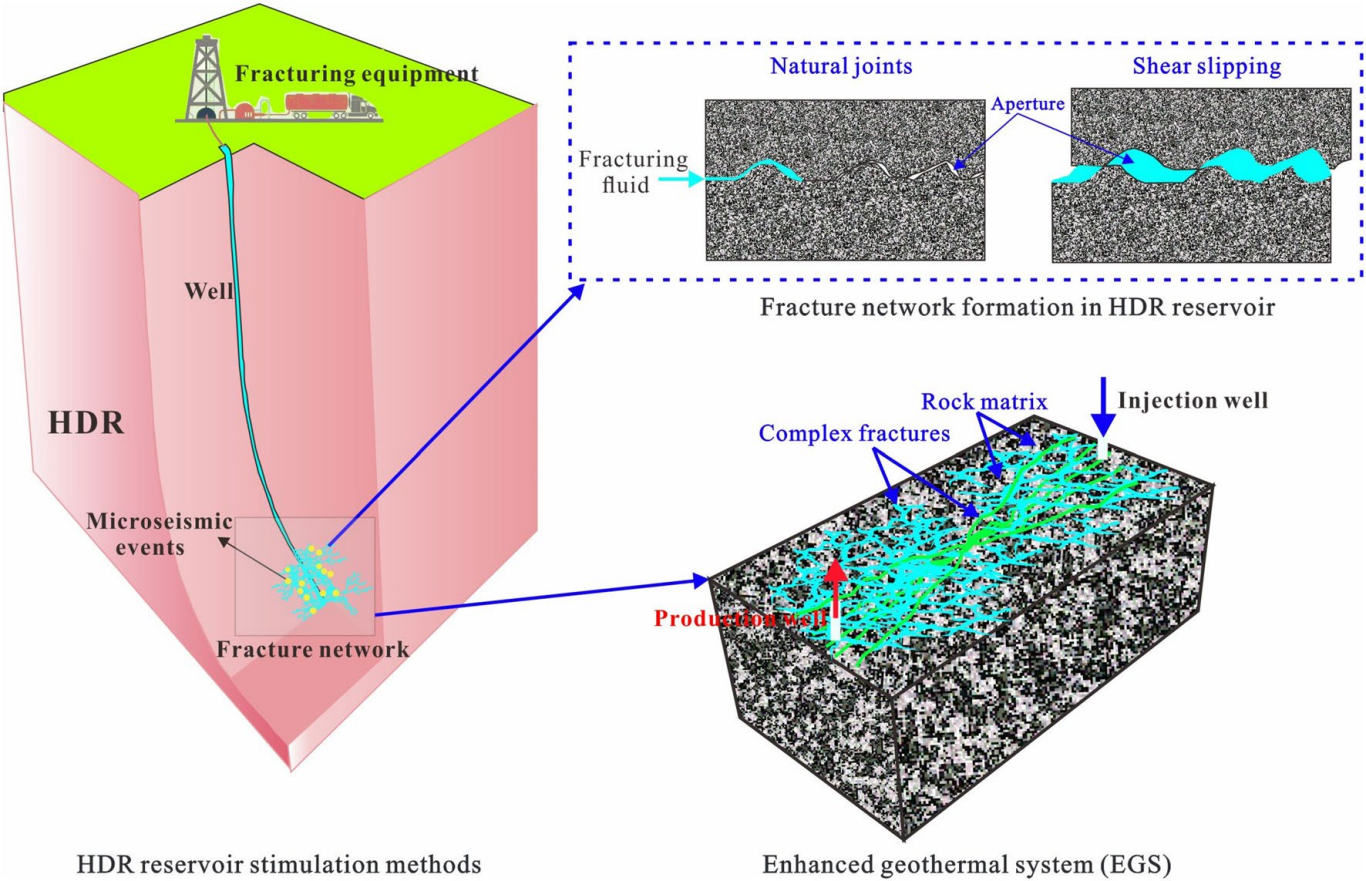
Schematic of enhanced geothermal system creation.

Widely used methods to enhance HDR permeability are currently hydraulic, chemical, and thermal stimulation techniques. Wherein hydraulic simulation is employed to develop HDR project worldwide with varying successes. Hydraulic stimulation treatments can be divided into tensile stimulation (gel-proppant fracturing) and shear stimulation (water fracturing) methods. Hydraulic shearing stimulation is currently the most popular method for improving HDR permeability, which involves opening natural fracture networks and/or creating new fractures by low injection pressure. The engineering process of heat extraction from these fractured hot rocks is called enhanced geothermal systems (EGS)^5, 7^. EGS has the advantage of capturing more abundant heat from the fractured geothermal reservoir, and many EGS projects have been developed worldwide^8^, e.g., Fenton Hill EGS in the United States^9^, Soultz-Sous-Forêts EGS in France^10^, Groß Schönebeck EGS in German^11^, etc. Such an enhanced geothermal system generally contains three media: rock matrix, natural fractures, and hydraulic fractures, as shown in Fig. 1.

In EGS, the fracture network geometry, fracture length, fracture aperture, and fracture orientation, etc*.*, are all unpredictable because of the complex deep environment. The fracture network composed of natural and hydraulic fractures is potential flow channels where the migration of the injected working fluids preferentially concentrates along the connected fractures between the injection and production wells, and the local heat is carried by these working fluids from the hot rocks to the wellhead for human needs^12, 13^. Currently, there are three methods for representing fractures in the EGS reservoir: (1) equivalent porous model^14, 15^; (2) dual porosity model^16–18^; and (3) discrete fracture network (DFN) model^12, 19–21^. The two former models generally consider some idealized fracture networks with simplified geometry, which cannot fully reflect heterogeneity and randomness of fractures in EGSs; while modelling DFN can explicitly characterize fracture parameters, such as location, aperture size, and orientation, etc. Moreover, DFN approach enables the representation of the behavior of individual fractures in reservoirs, which in turn reflects in the macroscopic responses of the system^22^. As a result, modelling DFN model can help us better understand the important behaviors of flow, heat transfer, and mechanics in fractured reservoirs. Under the framework of DFN model, many scholars have investigated the impact of fracture parameters on the thermal extraction performance of EGS. Xie et al.^23^ established a 3D fractured reservoir model to study the impact of various fracture parameters, such as randomness, groups, numbers, crossing angle, strike pitch and geometry of fracture networks on the production performance of EGS. Luo et al.^24^ studied the influence of the fracture surface roughness on fluid flow and heat transfer based on a DFN model. Shi et al.^25^ investigated the influences of fracture parameters, including number of fractures, fracture length and orientation, on multilateral-well EGS performance based on a DFN model. Yao et al.^26^ developed a 3D discrete fracture network model to evaluate heat production performance of the Desert Peak EGS project. Qu et al.^27^ found that complex fracture networks based on DFN model are beneficial to the thermal extraction form EGS. Therefore, DFN model is an important method for studying the impact of fracture parameters on the heat extraction performance of EGS. Here, we establish a 3D DFN model to investigate the geothermal energy mining potential of the Gonghe Basin in Northwest China, which could provide valuable insights for the fracturing operation involved in EGS construction.

During the EGS injection-production process, when the cold working fluid starts to be injected into the fractured geothermal reservoir, stress disturbance occurs to accommodate the transient fluid pressurization and thermally induced stress, which results in EGS performances being affected by complicated thermo-hydro-mechanical (THM) coupling processes^12, 13, 28, 29^. Hicks et al.^30^ numerically investigated the impacts of stress perturbations in a HDR geothermal system found that the THM coupling can result in faster temperature drawdown than that of TH coupling. In addition, stress disturbances may also lead to changes in fracture parameters, thereby affecting the thermal extraction performance of EGS. Zhang et al.^12^ developed a fully-coupled THM model and employed a comprehensive range of evaluation indices to quantify the impact of fracture shear dilatancy on the long-term heat production performance of EGSs. Their results confirmed that the thermal production rate may be underestimated if the fracture shear dilatational behavior is not incorporated. Furthermore, the THM multifield coupling effect may involve many engineering problems, e.g., reservoir rock mass instability, induced micro-earthquakes^31, 32^ and working fluid loss^9^ as well, which has seriously affected the development vision of deep geothermal. Hence, to accurately quantify and optimize the heat extraction processes in EGSs, it is crucial to develop a computationally efficient DFN model considering fully-coupled THM behaviors.

In addition, some scholars also proposed different EGS designs attempting to exactly maximize heat extraction from the fractured hot rocks. At present, the EGS design mainly includes two aspects: well arrangement and injection parameters. The basic designs typically involve doublet or triplet systems that consist of one or two production wells and one injection well, aimed at achieving a water circulation system^11^. Multi-well designs incorporating hexagonal or five-spot patterns of vertical wells can effectively enlarge the heat transfer area^11, 33^. In addition, some scholars have proposed new well arrangements for EGS designs, such as Zeng et al.^34^ proposed a novel double horizontal wells EGS to study the thermal extraction performance in Desert Peak geothermal site; Song et al.^35^ proposed a novel EGS with multilateral wells which could use one main wellbore to achieve injection and production simultaneously. In terms of investigating the injection parameters, Xu et al.^5^, Lei et al.^36^ and Zhang et al.^37^ studied the effects of injection temperature and water injection rate on the thermal extraction performance of EGS. Based on the existing research foundation, this study focuses on the use of a classic double vertical well EGS to investigate the thermal extraction performance of a complex fracture network under different injection parameters.

For the fracture network EGS reservoir, the strategies for evaluating the thermal extraction performance have been investigated by identifying the effect of various parameters on EGS performance^22^. The thermal performance parameters mainly include production water temperature (*T*_*pro*_), electrical power (*W*_*e*_), and thermal output power (*W*_*h*_), etc. In this work, we select production temperature, and thermal output power as the indicators to evaluate the thermal performance of fractured geothermal reservoirs. Production temperature is defined as the average temperature of the hot water extracted from the production well^35^. Garnish et al.^5, 38^ proposed that production temperature drop in an economically successful EGS during its operation period should not exceed 10%. The output thermal power represents the net heat production rate of EGS, which is related to production temperature and fluid mass flow rate^5, 37^. Hydraulic parameters include injection pressure (*P*_*inj*_) and flow impedance (*I*_*R*_), both of which can reflect the fluid transport capacity in the fractured reservoir^33, 37^. The ideal condition is that *P*_*inj*_ should always be lower than the in-situ minimum principal stress in the reservoir to avoid instability of the reservoir rock mass^37^. In order to quantify the geothermal energy extraction efficiency, heat extraction ratio is defined as the extracted heat from the reservoir divided by the heat energy stored in the initial reservoir^35^. Zhang et al.^12^, Song et al.^35^ have used the heat extraction ratio to evaluate the thermal performance of EGS.

In conclusion, the major challenge of EGS stimulation modeling is still that the multi-physics coupling processes occur at various scales in a complex and discontinuous hot rock stratum. This study employs discrete fracture network (DFN) modeling, in which each fracture is assigned with independent geo-physical properties, including permeability, aperture size, and orientation, i.e. we treat a random fracture network. With DFN modeling, the behaviors of working fluid flow, heat transfer, and stress evolution in a fractured geothermal reservoir are investigated in great detail. Our research results and insights are expected to have important implications for the deep-seated geothermal geoengineering activities.

## Thermo-hydro-mechanical (THM) coupling model

### Equation of state for working fluid

In this study, water is used as a working fluid due to its convenience and favorable thermophysical properties. Under high pressure and high temperature conditions in deep-seated reservoir, the water density $\rho_{w}$ (kg/m^3^), viscosity $\mu$ (Pa·s), specific heat capacity $C_{w}$ (J/(kg K)) and thermal conductivity $\lambda_{w}$(W/(m K)) vary as a function of temperature, which can be described as a function of temperature as^39^:

1 $$\rho_{w} = 838.466 + 1.401T - 0.003T^{2} + 3.718 \times 10^{ - 7} T^{3} {\text{, T}} \in [293{\text{ K}},550{\text{ K}}]$$

2 $$\mu = \left\{ \begin{aligned} & 1.379 - 0.021T + 1.36 \times 10^{ - 4} T^{2} - 4.645 \times 10^{ - 7} T^{3} + 8.904 \times 10^{ - 10} T^{4} - 9.079 \times 10^{ - 13} T^{5} \hfill \\ & + 3.845 \times 10^{ - 16} T^{6} , \, T \in {[273}{\text{.15 K,413}}{\text{.15 K]}} \hfill \\ & 0.004 - 2.1075 \times 10^{ - 5} T + 3.8578 \times 10^{ - 8} T^{2} - 2.3973 \times 10^{ - 11} T^{3} , \, T \in [413.15{\text{ K}},553.15{\text{ K}}] \hfill \\ \end{aligned} \right.$$

3 $$C_{w} = 12010.15 - 80.41 \times T + 0.31 \times T^{2} - 5.38 \times 10^{ - 4} T^{3} + 3.62 \times 10^{ - 7} T^{4}$$

4 $$\lambda_{w} = - 0.869 + 0.009 \times T - 1.5{8} \times 10^{ - 5} T^{2} { + 7}{\text{.98}} \times 10^{ - 9} T^{3}$$

To simplify the numerical model, the water phase transition is not considered under current pressure and temperature conditions. We assume the fractured reservoir is fully saturated by a single-phase fluid before the working fluid is injected^19^. Thus, the flow of working fluid in such saturated fractured porous media could be described by Darcy's law. We consider hypothetically the surrounding rock outside the fractured reservoir as a nearly impermeable environment, so the working fluid loss of the rock beyond the boundaries of the fractured reservoir is neglected^5, 40, 41^.

### Governing equations

1. Fluid flow in the fractured reservoir
  The continuity equation for fluid flow in a fractured porous rock reservoir is given by:
  5 $$\frac{\partial }{\partial t}(\phi \rho_{w} ) + \nabla \cdot (\rho_{w} {\mathbf{U}}) = Q_{m}$$
  where $\rho_{w}$ is the density of the working fluid, kg/m^3^; $\phi$ is the reservoir porosity; $t$ is the time, s; ${\mathbf{U}}$ is the flow velocity, m/s; $Q_{m}$ is the mass flow from the rock matrix into fractures, kg/(m^3^ s). According to Darcy’s law, the flow velocity ${\mathbf{U}}$ in fractures is described by:
  6 $${\mathbf{U}} = - \frac{k}{\mu }(\nabla p + \rho_{w} {\mathbf{g}})$$
  where $k$ is the reservoir permeability, m^2^; $\mu$ is the dynamic viscosity of fluid working, Pa s;$p$ is the fluid pressure in the rock; $\nabla$ is the divergence operator and $\rho_{w} {\mathbf{g}}$ represents the hydrostatic pressure gradient caused by the gravity effect.
  Compared to fractures that are measured in meters for their height and length, hydraulic fractures have a width that is typically measured in millimeters. As a result, the flow of seepage fluid along the width in the internal fracture can be disregarded. The seepage fluid within the fracture is simplified as a two-dimensional flow along its length and height. Thus, the equation for fluid flow in discrete fractures is written as:
  7 $$d_{f} \frac{\partial }{\partial t}(\phi_{f} \rho_{w} ) + \nabla_{T} \cdot (d_{f} \rho_{w} {\mathbf{U}}_{f} ) = d_{f} Q_{m}$$
  where $d_{f}$ is the fracture aperture, mm; $\phi_{f}$ is the fracture porosity; $\nabla_{T}$ denotes the gradient operator restricted to the fracture’s plane; ${\mathbf{U}}_{f}$ is the working flow velocity in the fracture, which is also given by Darcy’s law:
  8 $${\mathbf{U}}_{f} = - \frac{{k_{f} }}{\mu }(\nabla_{T} p + \rho_{w} {\mathbf{g}})$$
  where $k_{f}$ represents the fracture permeability, m^2^. Note that the parameter $Q_{m}$ in Eqs. (5) and (7) indicate the fluid exchange between the rock matrix and fractures by means of “inter-porosity flow”^35^.
2. Heat transfer in a fractured reservoir
  The heat exchange between the reservoir rock and working fluid can be described by Eq. (11):
  9 $$(\rho C_{w} )_{eff} \frac{\partial T}{{\partial t}} + \rho_{w} C_{w} {\mathbf{U}} \cdot \nabla T - \nabla (\lambda_{eff} \nabla T) = Q$$
  where $(\rho C_{w} )_{eff} = (1 - \phi )\rho_{s} C_{s} + \phi \rho_{w} C_{w}$ is the effective volumetric heat capacity of the fluid–solid; $Q$ is the heat source (or sink); $\rho_{s}$ is the rock density, kg/m^3^; $Cs$ is the heat capacity of rock matrix, J/kg K; $T$ is reservoir temperature at a certain time, ℃; $\lambda_{eff} = (1 - \phi )\lambda_{s} + \phi \lambda_{w}$ is the effective thermal conductivity of the fluid–solid mixture, W/(m‧K).
  In EGS, the injected cold fluid exchanges heat with the hot surrounding rock on both sides of the fractures, thereby the transport of heat occurs faster in the fractures than that in the surrounding medium. The energy equilibrium for heat transfer in discrete fractures is governed by:
  10 $$d_{f} (\rho C_{w} )_{eff} \frac{\partial T}{{\partial t}} + d_{f} \rho_{w} C_{w} {\mathbf{U}} \cdot \nabla_{t} T - \nabla_{t} \cdot d_{f} (\lambda_{eff} \nabla_{t} T) = Q$$
  Here, the parameter $Q$ in Eqs. (9) and (10) suggest that the heat transfer occurs between the rock matrix and fractures, which results in the fluid flowing through the fracture to be heated^35^.
3. Stress field response
  In the injection-production production, the mechanical equilibrium of the reservoir rock takes into account the in-situ stress, thermal stress, and fluid pressure, which can be described as^12, 42^:
  11 $$Gu_{i,jj} - \frac{G}{1 - 2\nu }u_{j,ji} + \alpha p + K\alpha_{T} T_{i} + F_{i} = 0$$
  where $G = {E/{2(1 + \nu )}}$ is the shear modulus of the reservoir rock, in which $E$ (GPa) and $\nu$ are the elastic modulus and Poisson’s ratio of the reservoir rock; $Fi$ ($i = x,y,z$) is the body force component per unit volume, MPa; $u_{i}$ is the displacement in the *i*-coordinate, m; $p$ is the fluid pressure and $\alpha$ is Biot coefficient, so the term $\alpha p$ represents the fluid pressure inside fractured-porous media; the term $K\alpha_{T} T_{i}$ represents the thermal stress, in which $K = {{2G(1 + \nu )}/{3(1 - 2\nu )}}$ is the bulk modulus of the reservoir rock, $\alpha T$ is the thermal expansion coefficient of reservoir rock, and $T_{i}$ is rock matrix temperature at this time.
  The Mohr–Coulomb criterion is the most popular criterion in rock mechanics. The criterion states that failure occurs when the shear stress and the normal stress acting on any element in the material satisfy the equation:
  12 $$\frac{1}{2}(\sigma_{1} - \sigma_{3} ) + \frac{1}{2}(\sigma_{1} + \sigma_{3} )sin\varphi - c\cos \varphi = 0$$
  For this isotropic criterion, enter the cohesion c, and the angle of internal friction *φ*. The failure index is then computed from:
  13 $$f_{i} = \frac{{\sqrt {J_{2} } m(\theta )}}{{k - \beta I_{1} }}$$
  where $I_{1} = \sigma_{1} + \sigma_{2} + \sigma_{3}$ and $J_{2} = \frac{1}{6}\left[ {(\sigma_{1} - \sigma_{2} )^{2} + (\sigma_{2} - \sigma_{3} )^{2} + (\sigma_{3} - \sigma_{1} )^{2} } \right]$ are the first and second deviatoric stress invariant; $m(\theta ) = \sqrt{\frac{1}{3}} \left( {1 + \sin \varphi } \right)\cos \theta - (1 - sin\phi )cos\left( {\theta + \frac{2\pi }{3}} \right)$, in which $\theta (0 \le \theta \le {\pi/3})$ is the Lode angle; $k = c\cos \phi$ and $\beta = {{\sin \phi }/3}$. In this study, the safety factor *SF* of the fractured reservoir during injection-production process is defined as:
  14 $$SF = \frac{1}{{f_{i} }}$$
  Under the Mohr–Coulomb criterion, the fractured reservoir failure occurs when $SF \le 1$. In a self-propping fracture system, the fracture permeability under the action of the solid stress field is described as^23, 43^:
  15 $$k_{f} = k_{f0} e^{{ - {{(\sigma_{n} - \alpha p)}/{\sigma^{ * } }}}}$$
  where $k_{f0} = {{d_{f}^{2} }/{12}}$ is the initial fracture permeability, m^2^; *σ*_*n*_ is total normal stress, MPa; $\sigma^{ * }$ is set as − 35 MPa in our work.

### Definition of EGS performance evaluation parameters

Performance criteria are needed to evaluate EGS operation of the fractured porous reservoir. For long-term EGS operation, production temperature ($T_{pro}$), injection pressure ($P_{inj}$), the output thermal power ($W_{h}$) and extraction heat ratio (*ξ*) are introduced for evaluation. The temperature of the produced water is given by:

16 $$T_{pro} = \frac{{\iint_{S} {T(t)ds}}}{S}$$

where $S$(m^2^) denotes the total area of the production well section and $T_{\left( t \right)}$ (℃) is the temperature of production well at time $t$.

The output thermal power ($W_{h}$) represents the net heat production rate of the EGS and can be calculated by Eq. (17)^35^:

17 $$W_{h} = Q_{inj} C_{w} (T_{pro} - T_{inj} )$$

where $Q_{inj}$ (kg/s) is the injection rate and $T_{inj}$ (℃) represents the injection temperature of the working fluid.

In order to quantify the heat extraction efficiency, heat extraction ratio ($\xi$) is defined as the extracted heat from the reservoir divided by the geothermal energy stored in the reservoir domain, that is^35^:

18 $$\xi = \frac{{\iiint_{{v_{r} }} {\rho_{s} C_{s} (T_{i} - T_{(t)} )dv}}}{{\iiint_{{v_{r} }} {\rho_{s} C_{s} (T_{i} - T_{in} )dv}}}$$

where $v_{r}$ is the volume of the reservoir domain, m^3^; $T_{i}$ and $T_{(t)}$ are the initial reservoir temperature and reservoir temperature at time $t$, respectively.

## EGS model with random fracture distribution

### Physical model

In EGS, the major contribution to improve permeability is the shearing of natural and artificial fractures in a nearly impermeable environment^11^. The conventional approach for developing HDR involves creating stimulated fractures that connect double wells, such as Fenton Hill EGS^9^, Groß Schönebeck EGS^11^, and Rosemanowes EGS^44^, a scenario also used in this paper to investigate the role of fracture network in EGS.

**Figure 2:**
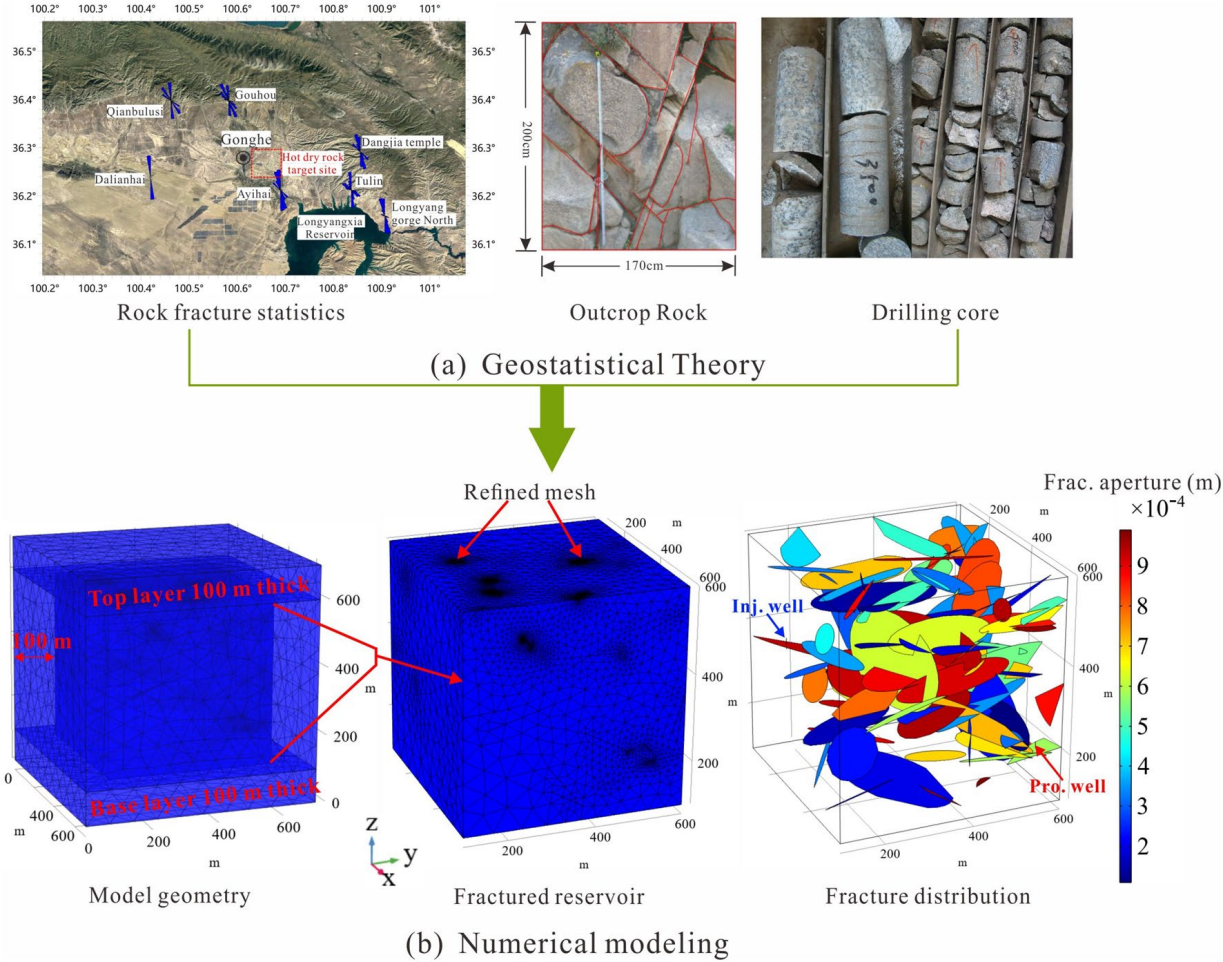
(**a**) Inference evidence for the target site fractured rock masses; (**b**) modeling of randomly fractured reservoirs (Base case).

Due to the established fracture systems are generally complex and random, modeling DFN helps to understand the characteristics of fluid flow and heat transfer in a fractured deep-seated geothermal reservoir. We consider hypothetically a 3D cube fractured rock domain of size *L* = 500 m as the thermal extraction zone, located at a depth of 3200–3700 m in granitic rock. The model depicted in Fig. 2a has dimension of 700 m × 700 m × 700 m, with the hypothetical fractured reservoir located at its center. The outside of the fractured zone is the intact and compact granitic surrounding rock. Figure 2b illustrates the grid subdivision of the reservoir region, with a particular emphasis on refinement at the location of the fracture. According to Fig. 2b, the injection well's central axis can be found in the fractured zone, ranging from (160, 160, 350) to (160, 160, 375), with the production well's axis located between (560, 560, 200) and (560, 560, 225).The elliptic fracture surfaces are created to connect the injection and production wells and their length, position, orientation, and aperture all follow a uniform distribution, the probability density function for the variable *x* is given by Eq. (16).

Our target HDR site, located in the Gonghe Basin in Northwest China, is an EGS project undergoing trial production. Evidence from drilling core samples and outcrop rocks from the target HDR site (Fig. 2a) indicates that there are numerous densely distributed natural fractures within the reservoir. Based on geological statistics, the natural fractures in the granite strata of the Gonghe Basin can be roughly classified into two dominant directions, NE and NW^33^. Since hydraulic fractures propagate parallel to *σ*_*H*_ but affected by natural fractures during hydraulic shearing simulation^45^, the created EGS should contains three media: rock matrix, natural fractures (NFs), and hydraulic fractures (HFs). During the process of hydraulic shear fracturing, the generation of HFs will connect with NFs to form a self-propping fracture network system that greatly enhances permeability. The study conducted by Lei et al.^36^ suggests that a well spacing design of 400–600 m is deemed appropriate for EGS in the Gonghe Basin. Considering the limitations of the hydraulic fracturing technology for HDR reservoirs, achieving communication between injection and production wells would require 3–5 fracturing treatment stages with such a well spacing design; moreover, the fracture network in such an EGS that contribute to heat extraction is composed of both natural and hydraulic fractures, thereby the fracture length is randomly set as 50–150 m in this work. This range of random fracture length was also used by Xie et al.^23^ to investigate the heat extraction performance of EGS. In addition, Kalinina et al.^46^ conducted a statistical study on EGS worldwide and found that the aperture size of self-propping fracture formed in granitic HDR reservoirs typically ranges from 0.1 to 1 mm, thus, the fracture aperture size in our DFN modelling is set as a random distribution ranging from 0.1 to 1 mm. All key geometrical parameters and reservoir properties are listed in Table 1, in which the porosity of the reservoir rock matrix is also purely random, i.e., statistically homogeneous. The probability density function for the variable *x* is given by:

19 $$f(x) = \left\{ \begin{array}{ll} \frac{1}{{x_{\max } - x_{\min } }} & \quad {\text{ for }}x_{\min } \le x \le x_{\max } \hfill \\ 0 & \quad {\text{ else}} \hfill \\ \end{array} \right.$$

**Table 1:** Reservoir rock and fracture properties.

Parameter	Value
Reservoir rock density, *ρ*	2700 kg/m^3^
Reservoir rock porosity, *ϕ*	0.001–0.03
Thermal conductivity of rock, *λ*_*s*_	3.0 W/(m K)
Specific heat capacity of rock, *C*_*s*_	900 J/(kg K)
thermal expansion coefficient of rock, *α*_*T*_	4e−7 1/K
Rock matrix permeability, *k*	1e−15 m^2^
Elastic modulus of rock, *E*	50 GPa
Poisson’s ratio of rock, *ν*	0.21
Rock cohesion, *C*	30 MPa
Fracture cohesion, *C*_*f*_	0.5 MPa
Angle of internal friction of rock, *φ*	45°
Friction angle of fracture, *φ*_*f*_	40°
Biot coefficient, *α*	0.9
Fracture aperture, *d*_*f*_	0.1–1 mm
Fracture length, *L*_*f*_	50 m–150 m
Injection mass rate, *Q*_*inj*_	40 kg/s
Injection fluid temperature, *T*_*inj*_	20 ℃

### Initial and boundary conditions

**Figure 3:**
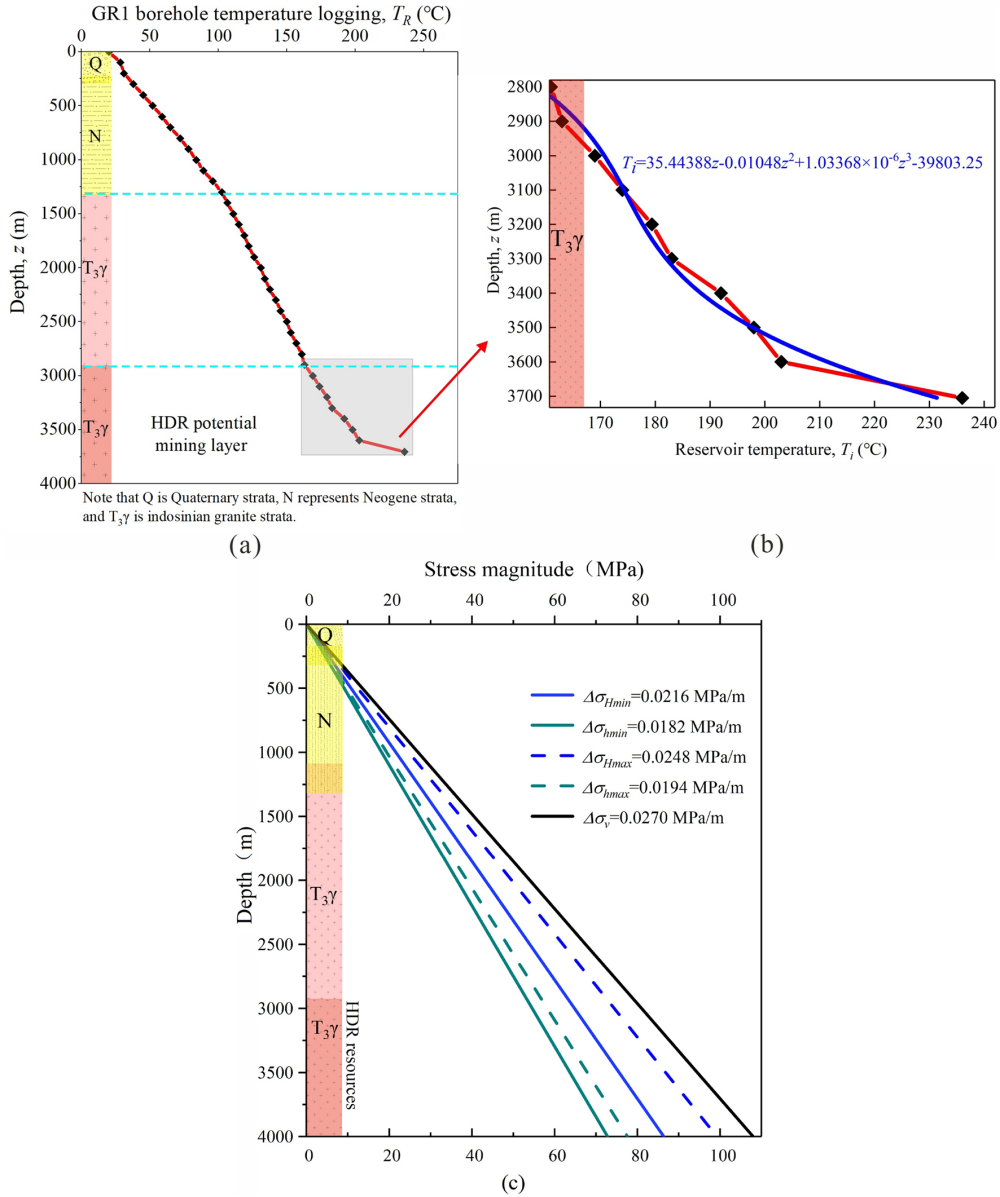
Formation temperature (**a**,**b**) and stress (**c**) variation with depth in the Gonghe Basin.

At present, four HDR Wells are drilled in the geothermal site, namely, DR3 (180.27 °C/2927.2 m), DR4 (178.72 °C/3102 m), GR1 (236 °C/3705 m), and GR2 (180 °C/3000 m), in which GR1 borehole is the most successful geothermal drilling in the Gonghe Basin, its formation temperature varies with depth as shown in Fig. 3a,b^36^. According to the latest scientific advances, the EGS pilot production project is expected to be constructed at a depth of nearly 4000 m in the Gonghe Basin. Thus, we assume that the geothermal reservoir is located at a depth of 3500–4000 m, where the formation temperature as a function of the depth in well GR1 as the initial temperature for our model. We consider hypothetically the fractured reservoir is in a water saturation state before the working fluid is injected, so the initial pore-water pressure gradient is set as 0.01 MPa/m.

Given the considerable impact of stress effects on the heat extraction form HDRs, it is therefore important to ascertain the in-situ stress field in the target formation. Numerous pieces of information, including in-situ stress measurements, fault analysis, and focal mechanism solution, imply that the maximum horizontal principal stress (*σ*_*H*_) in the target HDR site is dominant in NNE–NE directions^33, 45^. Considering the effects of the overburden's gravity and tectonic activity, Lei et al.^45^ proposed that the stress regime in the target geothermal formation is a normal faulting condition *σ*_*v*_ > *σ*_*H*_ > *σ*_*h*_, in which the estimated maximum principal stress (*σ*_*H*_) gradient and minimum principal stress (*σ*_*h*_) gradient are 0.0241–0.0248 MPa/m and 0.0188–0.0194 MPa/m, respectively. In addition, the vertical stress is estimated using the formula *σ*_*v*_ = *γh*, where *γ* = 27 kN/m^3^. In this way, they estimated that the *σ*_*H*_ is 77–92 MPa, and the *σ*_*h*_ is 60–72 MPa, and the *σ*_*v*_ is about 90–100 MPa in the target reservoir (Fig. 3c). Thus, the established DFN model is subject to a stress state with the maximum horizontal stress *S*_*H*_ = 80 MPa, the minimum horizontal stress *S*_*h*_ = 67 MPa, and the vertical principal stress *S*_*v*_ = 90 MPa, applied along the face perpendicular to the *x*, *y* and *z* directions, respectively. In addition, since hydraulic fractures propagate parallel to *σ*_*H*_ but affected by natural fractures during hydraulic simulation, we here arrange the production well and injection well in the direction of the angle bisector of the *x-* and *y-*axes, which ensures consistency with the actual on-site conditions.

### Characteristics of coupled THM processes in fractured reservoir

**Figure 4:**
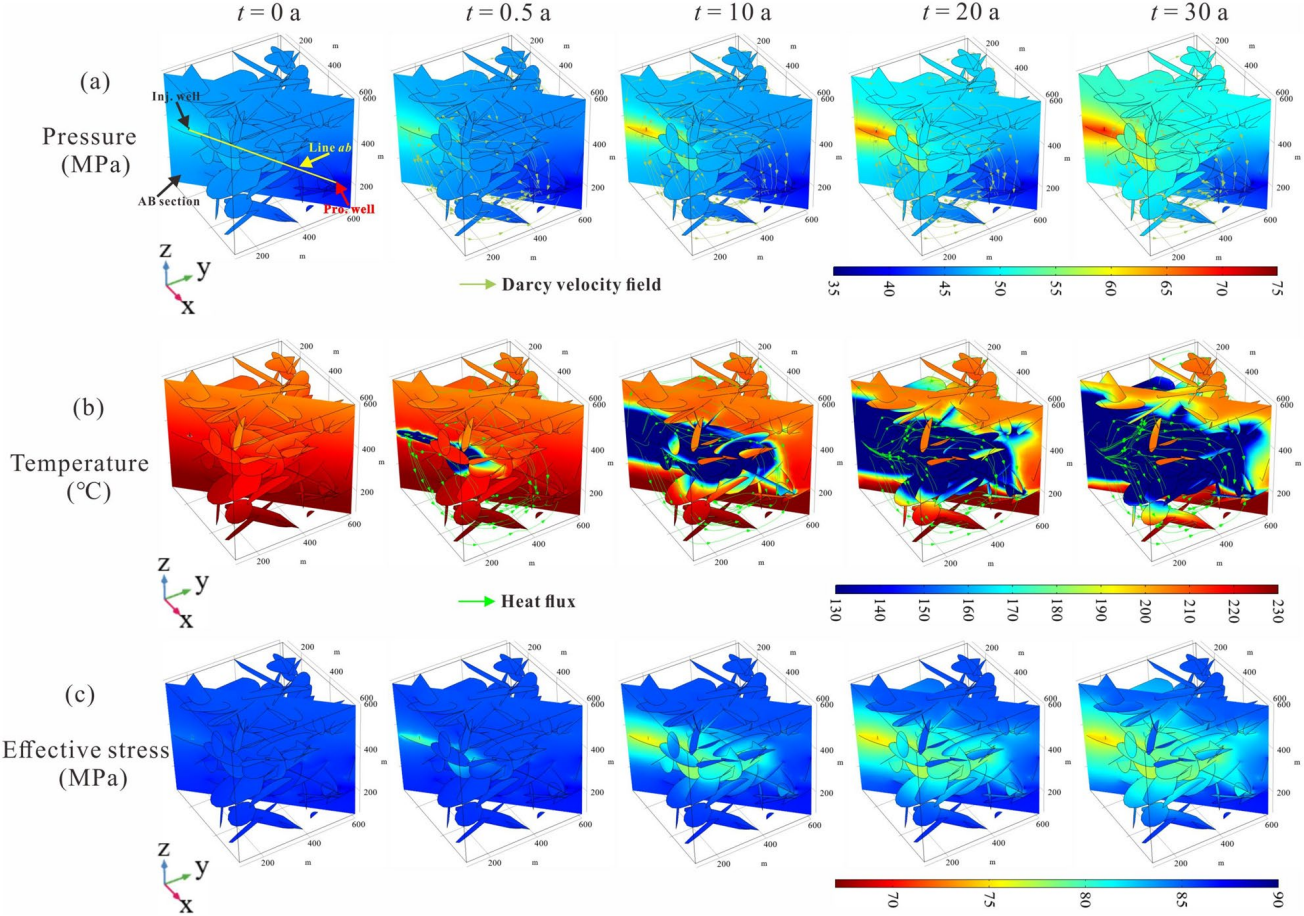
(**a**) Fluid pressure, (**b**) reservoir temperature, and (**c**) stress variation in the fractured rock during injection-production process.

Figure 4a,b illustrates the temporal and spatial variation in fluid pressure, reservoir temperature, as well as their streamlines in a 30-year operation time. The *AB* section connected injection and production wells is used to describe the temperature variation along the fluid flow path. At the beginning (*t* = 0 a), the rock domain is in its initial equilibrium state, where the pressure, temperature, and stress fields are homogeneous on the same horizontal plane. When the low-temperature working fluid starts to be injected into the fractured rock (*t* = 0.5 a), fluid pressure firstly accumulates in the fractures that originally connect to the injection well. The elevated pore pressure then drives the working fluid migration to the production well. Noted that a small amount of fluid diffuses into the low-permeability rock matrix, thus causing the pore pressure in the local rock matrix to rise. However, the high pressure mainly concentrates in the locally interconnected fractures, which indicates the working fluid mainly flows through a limited path between injection and production wells. The cryogenic fluid flows through the fractures and carry heat from the high-temperature rock to the production equipment, thereby the temperature around these fractures that originally connect to the injector drops first. As the EGS operation continues through at *t* = 5.0 a, the pressure near the injection well increases quickly to about 64 MPa, after which the cooled front reaches the fractures directly connected with the production well. In the 30th year, the cooled zone has spread into the rock matrix near the production well, which means that the EGS system will not operate efficiently. In summary, we can infer that both fracture and matrix are important roles for heat exchange in a fractured porous rock reservoir, but the connected fracture cluster between injection and production wells determines the flow path of the working fluid.

As the development of geothermal reservoirs by transient cooling proceeds, the solid stress perturbation occurs to accommodate transient fluid pressurization and thermal stress changes. Figure 4c depicts that low stress generally occurs in the region where high fluid pressure appears and is located in the center of the cooling zone, which is attributed to the poroelasticity effect. Compare the propagation rate of the three field disturbance ranges in the *xy* plane (horizontal plane), the fastest propagation rate is found in the temperature field, with the stress field ranked second, followed by the pressure field; however, in the *z-*direction (depth variation), the pressure field shows the largest disturbance range at any time. No matter which direction, the disturbance range of the solid stress field is always the smallest.

**Figure 5:**
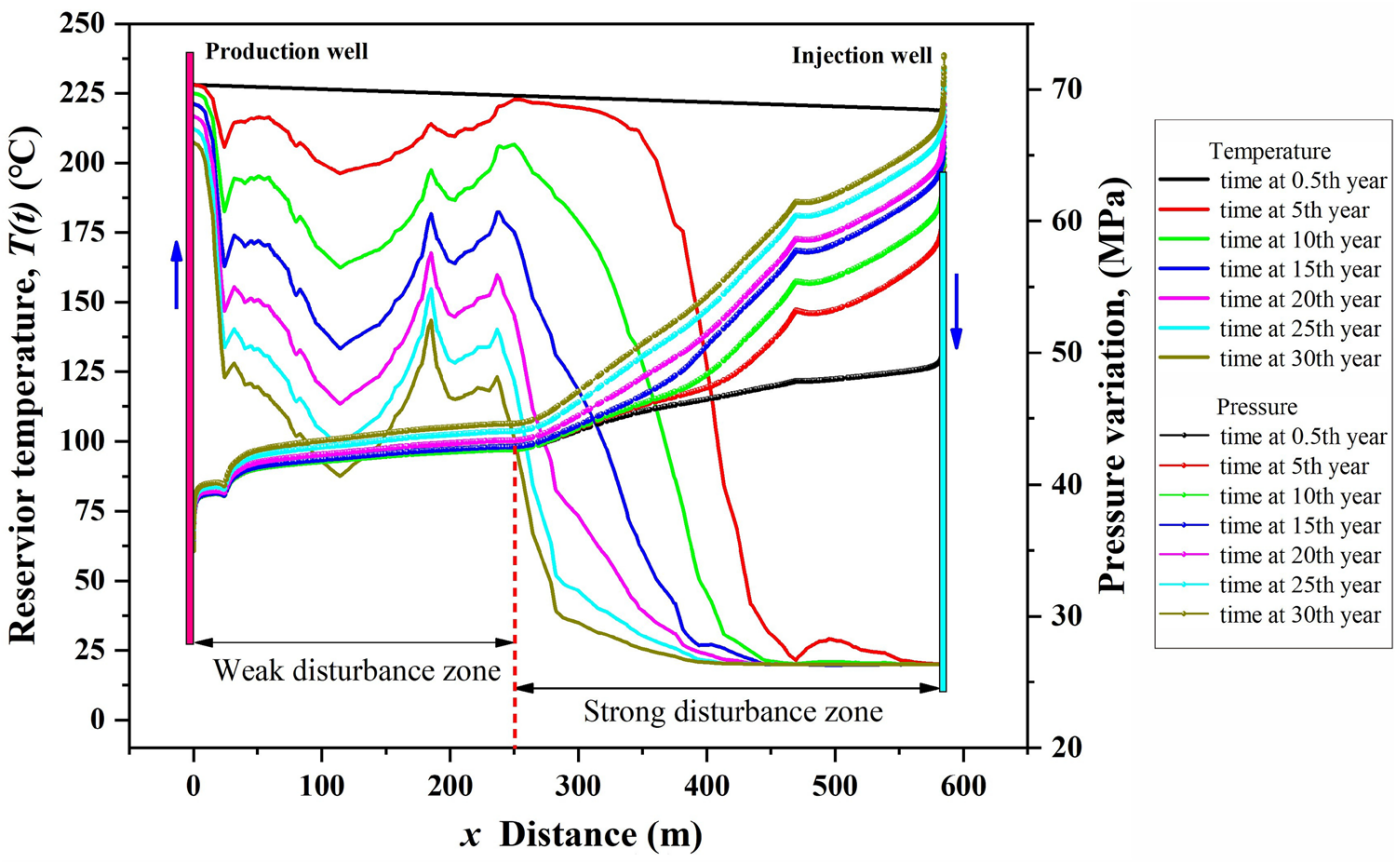
Temperature and pressure at different locations between injection-production wells vary with time.

Figure 5 presents the pressure and temperature distributions along line *ab* (from injection point to production point) at various times. The spatial fluctuations in the temperature and pressure curves are due to the fracture effect^35^. It can be observed that the temperature field is more sensitive to the fracture effect than that of the pressure field. When water is injected into the fractured rock (*t* = 0.5 a), the fluid pressure at *x* = 585.24 m (location of the injection point) rapidly rises to 52.72 MPa. As time goes on, the pressure shows an apparent upward trend within 5 years, an increase of about 12 MPa, after which the injection pressure slightly increases at a rate of 0.88–2.37 MPa every 5 years. Approximately 300 m away from the injection well, the increase in fluid pressure over time in the reservoir is negligible. As far away from the injection well, the increase in pressure over time diminishes. The pressure in the local domain of *x* = 50–250 m remained almost constant at 41–45 MPa during the entire operation time. Within a 50 m zone around the production well, the fluid pressure decreases significantly as the distance from the production well decreases but does not change much over time.

Affected by fluid flow, the rock domain at *x* = 250–585.24 m is dramatically cooled to below 120 °C in the 15th year. However, the rock temperature decreases slowly over time during the entire operation period in the local domain at *x* = 0–250 m, where is considered a potential range for cryogenic fluids to be heated to achieve geothermal extraction. From the above discussion, we believe that there is a strong disturbance zone of temperature and fluid fields around the injection well in the EGS production process. In this study, the strong disturbance zone is roughly within 335 m around the injection well.

## Results and discussion

### Influence of fracture network characteristics on EGS performance

#### Fracture network morphology

**Figure 6:**
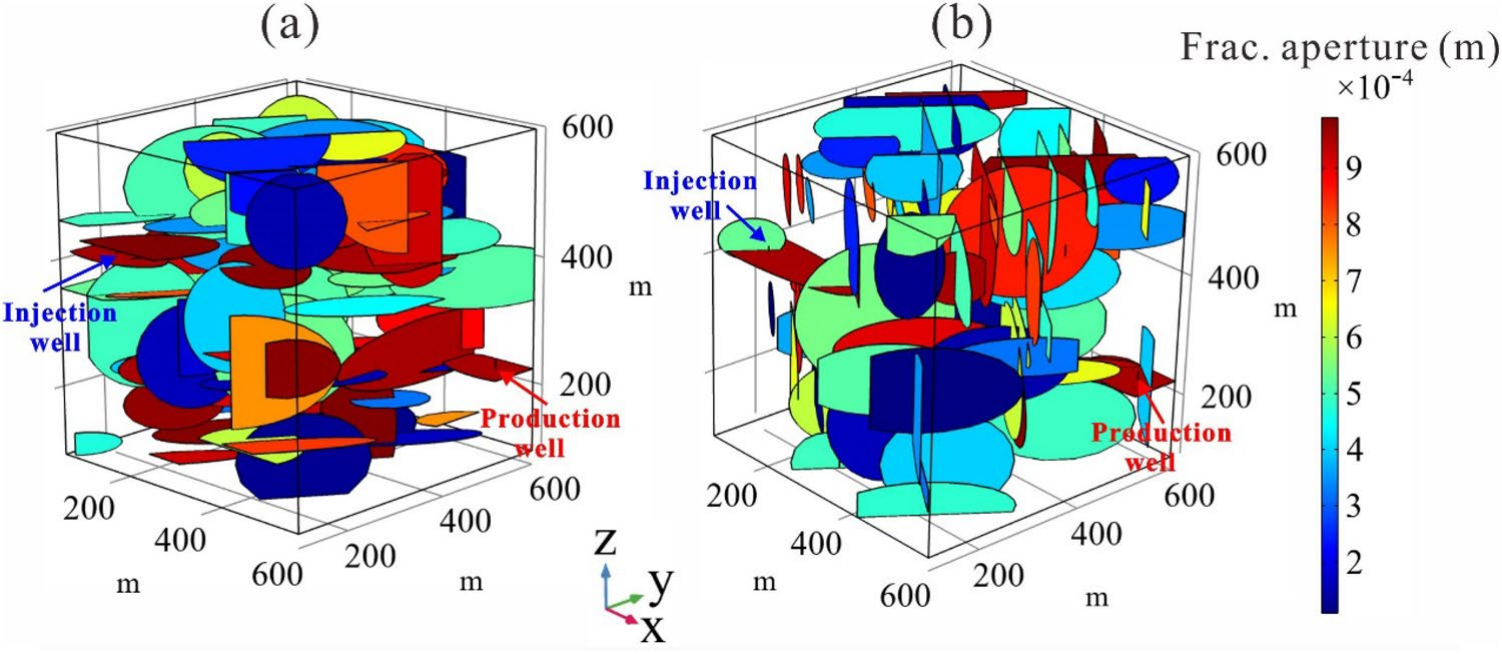
Different fracture network models for numerical simulations, (**a**) Model A consists of horizontal and vertical sets of mutually orthogonal fractures, and (**b**) Model B consists of two sets of mutually orthogonal vertical fractures.

To better unravel the role of fracture network in the THM processes in EGSs, we also generate two other networks with two orthogonal sets of fractures oriented at different orientations. As shown in Fig. 6, the fracture network model A is composed of a set of vertical fracture oriented at 125° with respect to the positive x direction and a set of horizontal fracture; Model B is also embedded with a network of 100 vertical fractures containing two sets oriented at 125° and 215° with respect to the positive x direction. In Model A, a set of horizontal fractures is introduced, and the resulting differences in the fracture network structure will facilitate further investigation into the impact of fracture dip on EGS performance. This is because the Gonghe Basin is located in the hub area of the subduction of the Indian Ocean plate to the Eurasian plate, the structural stress field has a greater influence on the in-situ stress field. Based on this, we speculate that the three principal stress axes of such a stress field are probably inclined, which may result in the creation of different fracture planes with varying inclination during hydraulic fracturing. The length and aperture of each set of fractures in Model A and Model B obey the uniform distribution.

**Figure 7:**
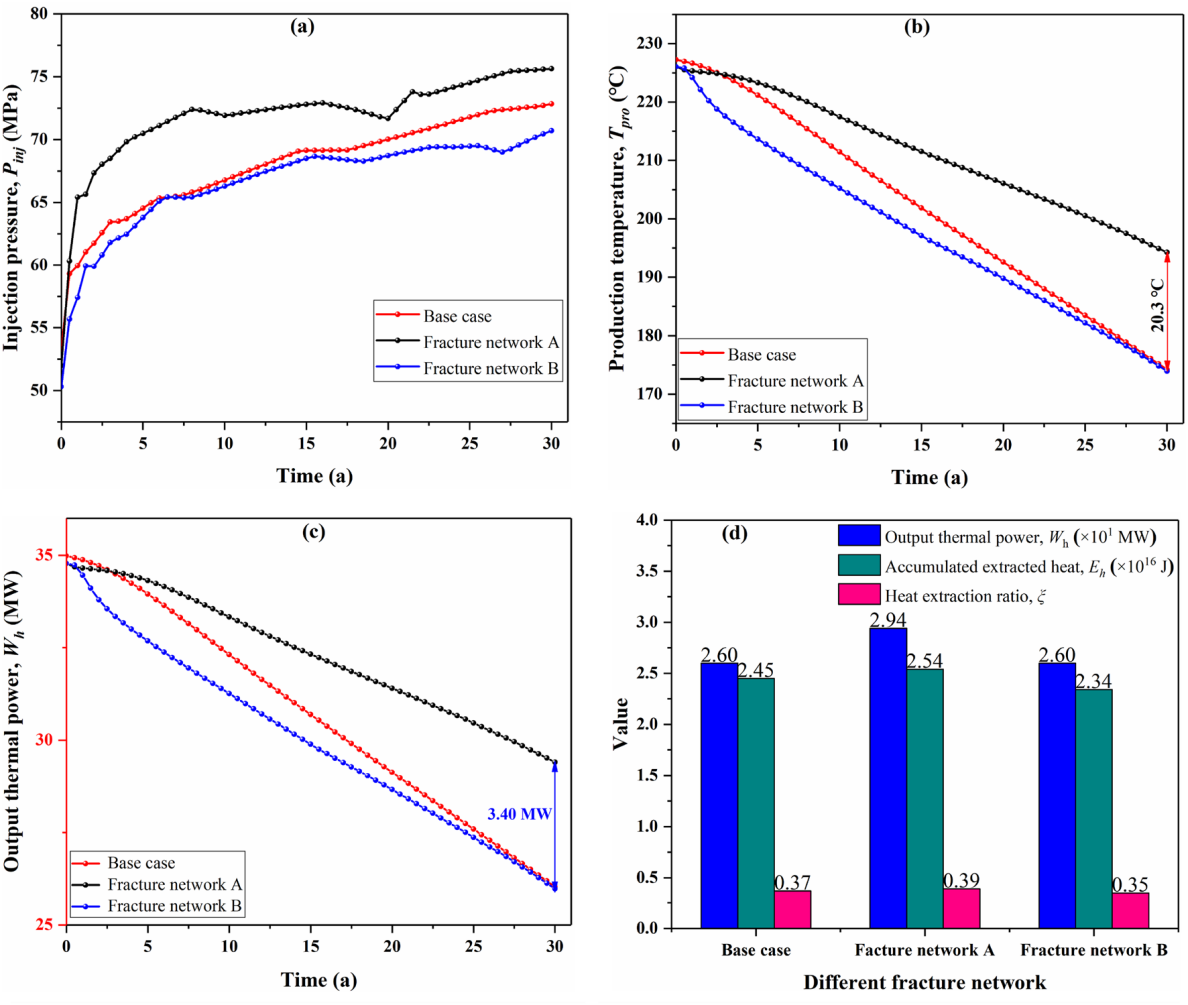
EGS performances of different fracture network models, (**a**) injection pressure, (**b**) production temperature, (**c**) output thermal power variation with operation time, and (**d**) the comparison of accumulative thermal energy, heat extraction ratio, and output thermal power after 30 years.

The fracture network determines the flow path of the working fluid in the fractured reservoir and thus affects the characteristics of fluid flow in EGS system. Here, we investigate the impact of fracture network morphology on EGS performance using identical injection parameters (40 kg/s injection rate and 20 °C injection temperature). As can be seen in Fig. 7a, the injection pressure curves exhibit a strong fluctuation upward trend with time. The increase in injection pressure with time is mainly caused by the increase of the viscosity of the working fluid; because the viscosity of the working fluid in our study is assumed to be a function of temperature, the viscosity of the working fluid increases gradually with the decrease of reservoir temperature. According to Darcy's law, the increase of fluid viscosity requires raising the injection pressure to maintain the constant flow of EGS. The strong temporal fluctuation phenomenon in injection pressure is considered related to the fracture distribution which leads to alternating occurrences of pressure build-up and depletion^47^. Under the condition of the same injection mass flow rate, the fracture network A requires a slightly higher injection pressure, with Base case ranked second, followed by fracture network B.

The fluid flow in different fracture models is different, which results in the difference of temperature field evolution in the reservoir domain and thus affects the production performance of EGS. As shown in Fig. 7b, *T*_*pro*_ of Base case is slightly higher than that of fracture network B with time, while *T*_*pro*_ of the fracture network A is much higher than that of the base case a and fracture network B in a 30-year time. At the end of the operation period, *T*_*pro*_ of Network A is 194.25 °C, which is about 20.3 °C higher than the other two models. Figure 7c depicts the output thermal power of the present considered cases. As time proceeds, the thermal output power of each network model decreases continuously with its production temperature. The thermal output power of Network A is always higher than that of the other two cases due to its better production temperature performance. The difference of thermal output power increases gradually with time, and at the end of operation, the output power of Network A is about 3.40 MW higher than that of the other two cases. The output thermal power of the Base case is slightly higher than that of Network model B because its slightly higher *T*_*pro*_ within 30 years. Figure 7d presents the comparison of the output thermal power, accumulated extracted heat energy, and heat extraction ratios of the presently considered models at the end of operation time. The accumulated extracted heat energy and heat extraction ratio of the Network A are 2.54 × 10^16^ J and 0.39, respectively, in a 30-year operation period, showing the best geothermal extraction performance.

**Figure 8:**
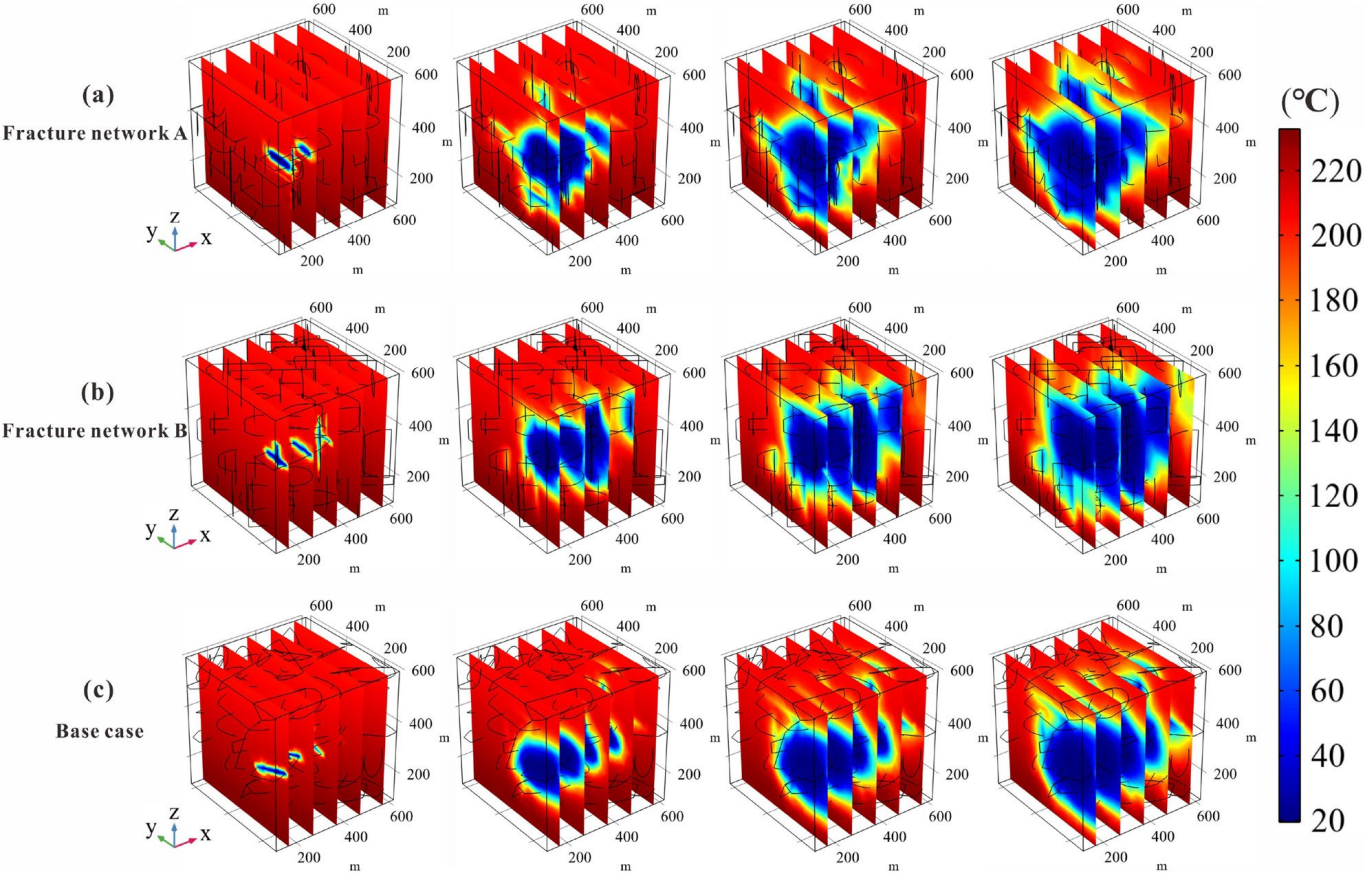
Temperature field evolution of different fracture network models.

To further determine the reason for the difference in the production performance of different fracture networks, we investigate the temperature distributions in the fractured reservoir of the three cases, as shown in Fig. 8. It can be observed that the cooled rock domain in the *xy-palne* of the Network A extends significantly further than in the other two cases, while the cooled domain of Network B is more dominant in the *z*-direction. The cooled range of the Base case is always ranked second during the entire operation period, both in *xy-plane* and *z*-direction. The evolution law of the cooled contour with time in three cases determine that the production temperature of Network A is higher than that of the Base case and Network B. Therefore, we infer that the horizontal fracture in the Model A play an important role in seepage and heat transfer. To prove this point, the next step is to investigate the effect of fracture dip on EGS performance.

#### Fracture dip

**Figure 9:**
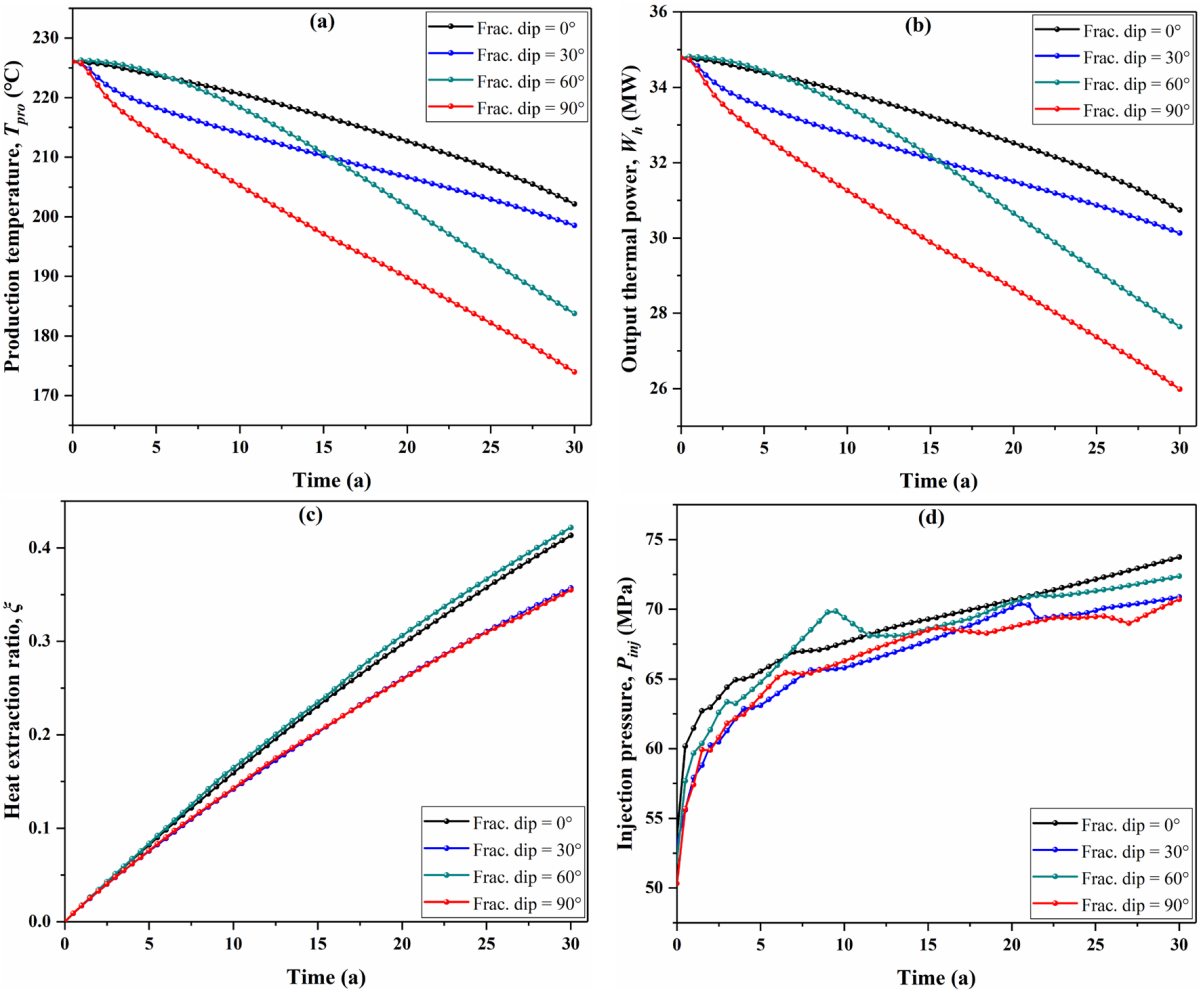
Effect of fracture dip on EGS performance, (**a**) production temperature, (**b**) output thermal power, (**c**) heat extraction ratio, and (**d**) injection pressure variation with time.

Figure 9a presents the variation of the temperature curve with time under different fracture dip scenarios. Within 15 years, the production temperature of the 60° dip scenario is higher than that of the 30° dip scenario or even the 0° dip scenario. After the 15th year, the production temperature in the 60° dip scenario starts to obviously below that in the 0° and 30° fracture dip scenarios, and its temperature decrease trend is roughly parallel to that of the 90° scenario. In a 30-year operation time, the production temperature in four different fracture dip scenarios decreased by 10.5% (from 226.05 to 202.16 °C), 12.2% (from 226.05 to 198.54 °C), 18.7% (from 226.05 to 183.76 °C) and 23.0% (from 226.05 to 173.95 °C) respectively. Figure 9b shows that the variation trend of output thermal power is similar to that of the production temperature. As a result, the fracture dip apparently influences the production temperature and output thermal power. Figure 9c depicts a comparison of the heat extraction ratio for four different fracture dip scenarios. It can be observed that the greatest heat extraction ratio is found in the 60° fracture dip scenario, with the 0° dip scenario ranked second, followed by the 30° and 90° scenarios. Figure 9d shows that the injection pressures in the four scenarios all fluctuate with time in the range of 50–73 MPa, thus, it can be inferred that the influence of fracture dip on the injection pressure was not prominent.

#### Fracture aperture

**Figure 10:**
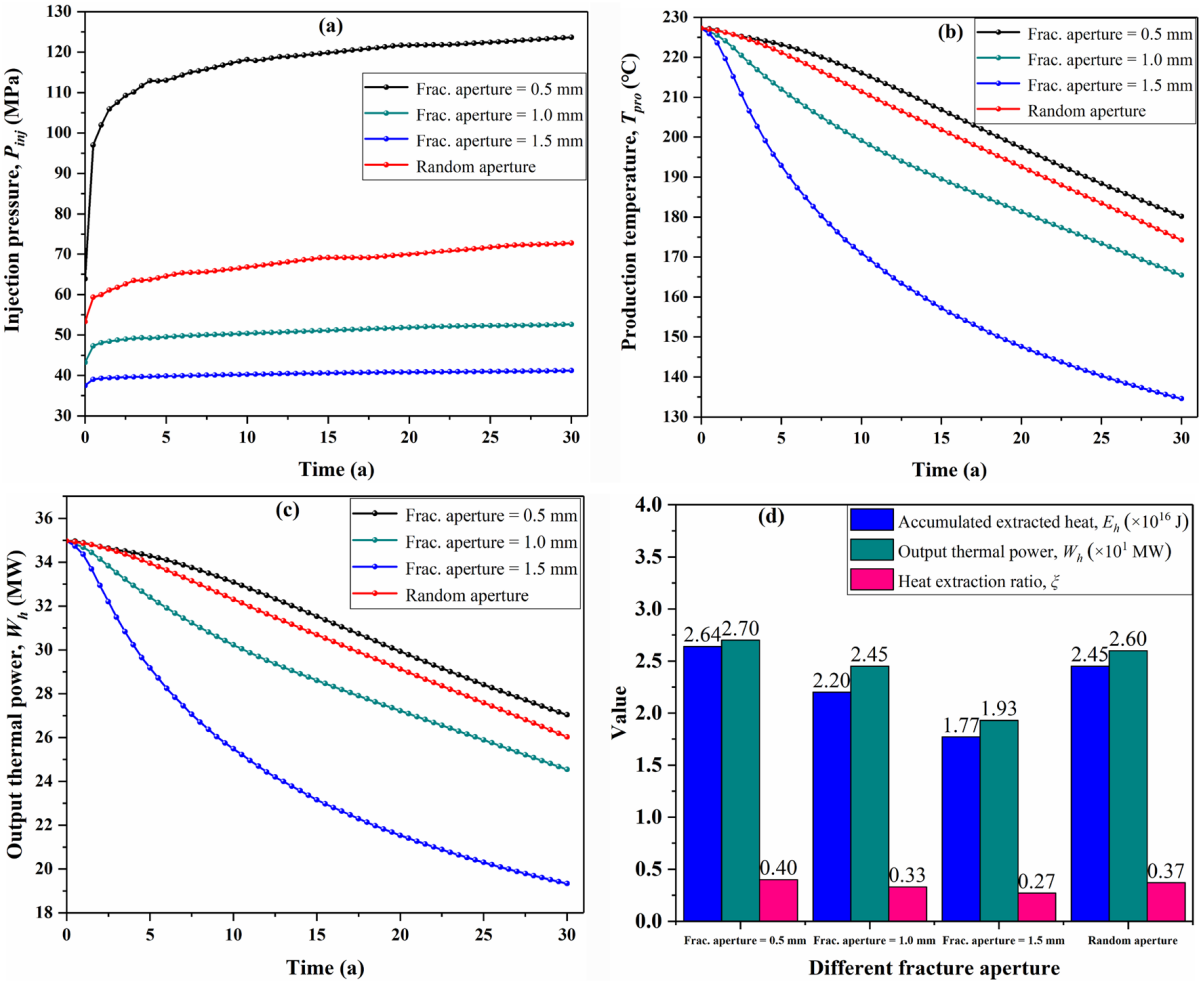
Effect of fracture aperture on EGS performance, (**a**) injection pressure, (**b**) production temperature, (**c**) output thermal power variation with time, and (**d**) the comparison of accumulative thermal energy, heat extraction ratio, and output thermal power after 30 years.

In a self-propping fracture system, the fracture aperture highly determines the flow behavior of the working fluid, thereby may affect EGS performance. Herein, we investigate the differences in production temperature, injection pressure, output thermal power, and heat extraction ratio over a 30-year period for four cases with 0.5 mm, 1.0 mm, 1.5 mm and randomly distributed aperture (the aperture range is 0.1–1.0 mm). The response of injection pressure to the variation of fracture aperture is depicted in Fig. 10a. The injection pressure increases with time for each case, mainly caused by the increase of the working fluid viscosity (arising from the decreasing reservoir temperature). For the case with 0.5 mm fracture aperture, the injection pressure increases rapidly with time within 5 years, after which the injection pressure increases slowly with time; as the increase of fracture aperture, the time for rapid increase in injection pressure becomes shorter. Besides, it can be also observed that the case with smaller fracture aperture requires higher injection pressure to maintain the same injection flow rate of the working fluid at any time. In a 30-year operation time, the injection pressure of three cases with 0.5 mm, 1.0 mm, 1.5 mm fracture aperture ascended by 59.82 MPa (from 63.84 to 123.66 MPa), 9.42 MPa (from 43.19 to 52.61 MPa), and 3.69 MPa (from 37.51 to 41.20 MPa) respectively. Thus, the EGS model with smaller fracture aperture shows the worse fluid injection performance.

It can be observed that the production temperature decreases significantly with the increase of fracture aperture (Fig. 10b). At the end of the operation time, the production temperature of the random aperture scenario decreases to 175.24 °C, which is 5 °C lower than the 0.5 mm aperture scenario but about 40 °C higher than the 1.5 mm fracture scenario. This significant change in production temperature can be reflected in the heat output efficiency of EGS. As shown in Fig. 10c, the variation trend of output thermal power is similar to that of production temperature. Thus, the output thermal power of EGS also decreases significantly with the increase of fracture aperture. Figure 10d compares the accumulated extracted heat energy (*E*_*h*_) and heat extraction ratio (*ξ*) in a 30-year period for the four scenarios. It can be observed that *E*_*h*_ and *ξ* of the 0.5 mm fracture aperture scenario are 2.64 × 10^16^ J, and 0.40, respectively, showing the first-rate geothermal extraction performance. As the fracture aperture becomes larger, the two production indicators *E*_*h*_ and *ξ* all decrease significantly, so the 1.5 mm aperture scenario has the worst production performance.

To sum up, there is a conflict between the heat production performance and the fluid flow behavior during EGS operation. On the one hand, the fractured reservoir with smaller fracture aperture shows the first-rate production temperature performance, which is conducive to increase the duration of EGS operation. On the other hand, the fractured reservoirs with small fracture aperture the worse is fluid injection performance and higher injection pressure is reuqired to sustain EGS functioning, which induce instability of the reservoir surrounding rock in practical engineering and may be prohibited in an EGS design. So, the optimal fracture aperture should be kept in a range of 0.5–1.0 mm in a self-propping fracture system.

#### Fracture length

**Figure 11:**
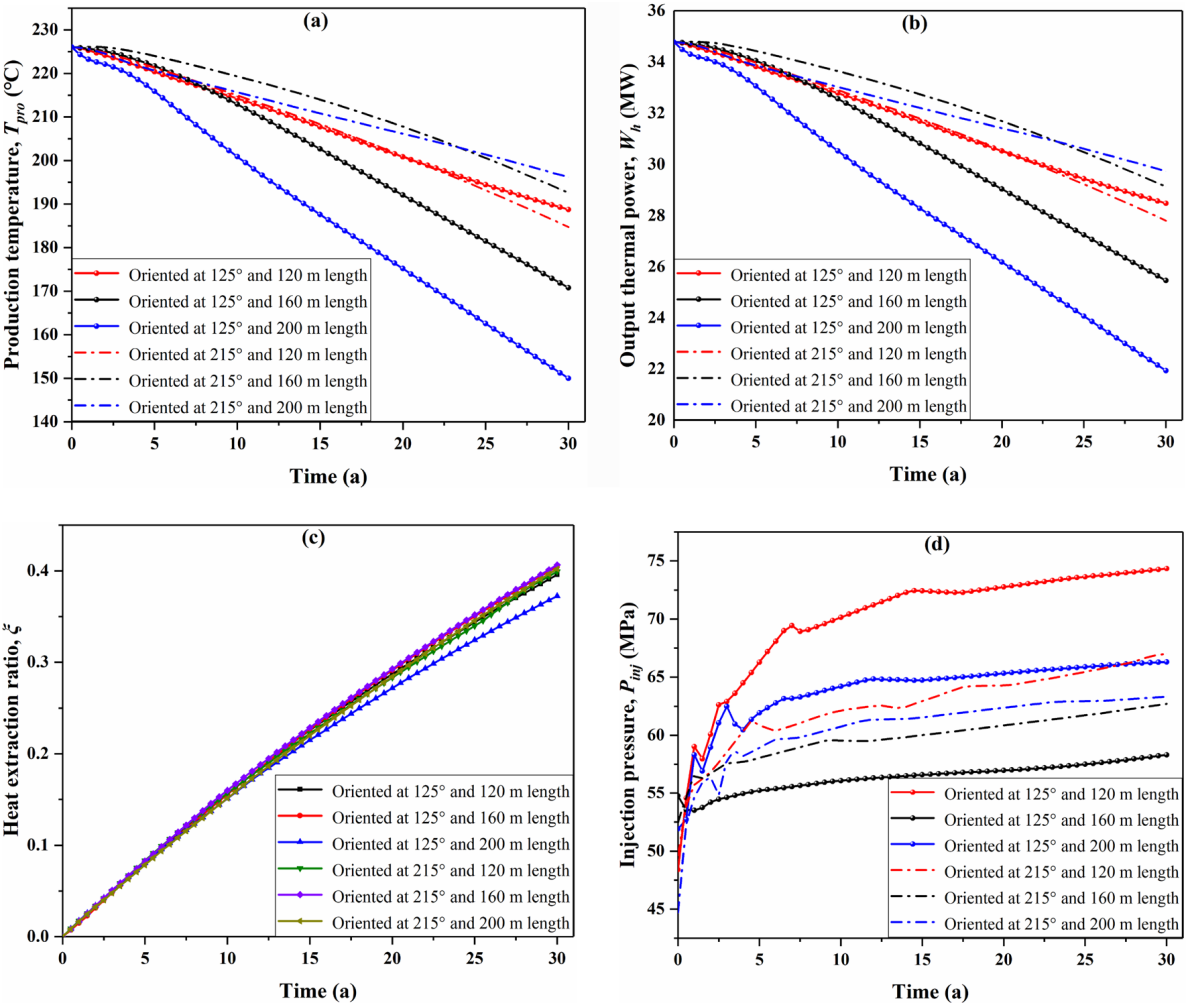
Effect of fracture length with different orientation on EGS performance, wherein the fractures oriented towards 125° are roughly parallel to the injection-production well connection and those oriented towards 215° are perpendicular to the injection-production well connection direction: (**a**) production temperature, (**b**) output thermal power, (**c**) heat extraction ratio, and (**d**) injection pressure variation with time.

The production temperature decreases significantly with the increase of fracture length toward the injection and production wells (oriented at 125°), and increases slightly with the increase of fracture length oriented at 215° (Fig. 11a). The results reveal that the fracture length oriented at 125° increases by 40 m, the production temperature drops by about 20 °C at the end of operation time; this is because that the fracture length increases towards the injection and production wells easily to form a preferential flow path and thus cause a thermal short circuit. However, the fracture length in the orthogonal direction increases by 40 m, the production temperature increases 4 °C to 8 °C at the end of operation time; this is because the increase of fracture length in the orthogonal direction lengthens the flow path of the working fluid in the reservoir and thus the fluid is sufficiently heated. Affected by the production temperature, the output thermal power (Fig. 11b) of the present considered cases have a similar tendency to the production temperature. As a result, the increase of fracture length toward the injection and production wells apparently influences EGS production performance and it is adverse influence. It can be observed form Fig. 11c that the heat extraction ratio of the case with fracture orientation of 125° and the fracture length of 200 m is the smallest, because its corresponding production temperature and heat output power are the worst. Certainly, changing the fracture length will also affect the injection performance, as shown in Fig. 11d. With the same fracture length increasing, the influence of 125° direction on the injection pressure is obviously greater than that of the 215° direction. For the two fracture orientations studied, the maximum injection pressure is the case with a fracture length of 120 m and the minimum injection pressure occurs when the fracture length is 160 m. Thus, the effect of fracture length on EGS performance highly depends on the fracture orientation, the excessive increase of fracture length towards injection-production wells is detrimental to heat extraction.

### Influence of injection parameters on EGS performance

**Figure 12:**
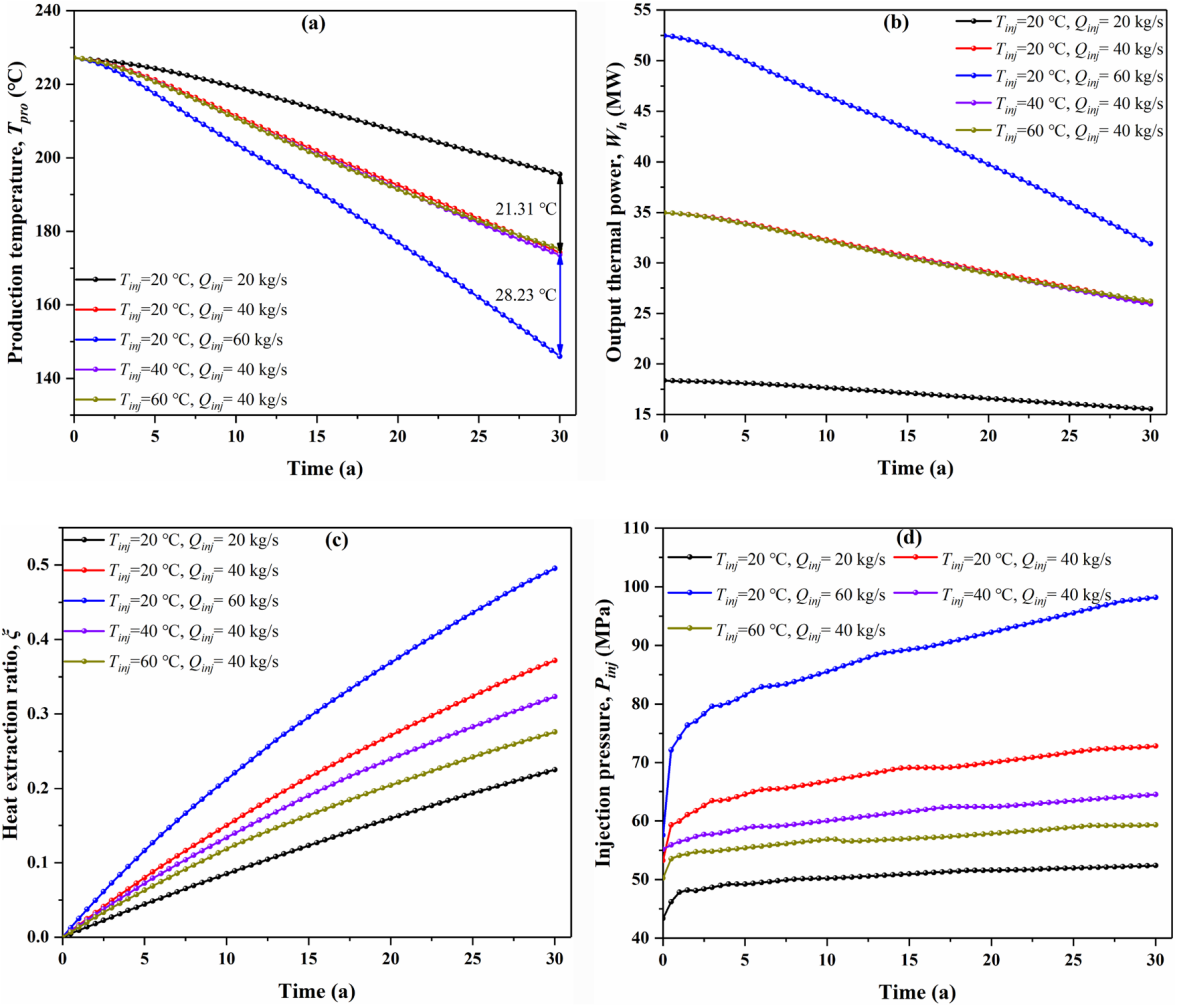
Effect of injection parameters on the production performance of random fracture model, (**a**) production temperature, (**b**) output thermal power, (**c**) heat extraction ratio, and (**d**) injection pressure variation with time.

The injection parameters affecting an EGS performance mainly include injection mass flow rate (*Q*_*inj*_) and injection temperature (*T*_*inj*_). The injection rate increases by 20 kg/s, the production temperature drops by over 20 °C under the same fracture network (Fig. 12a). However, when the injection rate is 40 kg/s, there is little production temperature difference as the injection temperature increases by 20 °C. As a result, the injection mass flow rate apparently influences the production temperature more than that of the injection temperature. It can be observed from Fig. 12b that the heat output power has a remarkable response to the injection mass flow rate, but little response to the injection temperature. Increasing the injection mass flow rate can significantly increase the thermal output power, but the disadvantage is that the downward trend of the thermal output power also increases significantly with the injection flow rate. The thermal output power at three different injection flow rates decreased by 15.3%, 24.7%, and 39.2%, respectively, in 30-year time, thus, the case with an injection rate of 60 kg/s shows the worst heat production performance. The geothermal extraction ratio increases with the increase of injection flow rate, but decreases with the increase of injection temperature (Fig. 12c). A high injection rate leads to a significantly high injection pressure, while increasing the injection temperature at the same injection rate can reduce the injection pressure (Fig. 12d). For a successful EGS, we expect to maintain high production temperature, high output power, high heat extraction ratio, and low injection pressure during the production period to safely and efficiently exploit geothermal energy. Hence, for a fractured porous EGS, we believe that the injection rate of 40 kg/s and an injection temperature of 60 °C are appropriate.

### Reservoir solid field response

In HDR mining process, fluid pressure, stress, and temperature fields are the important factors influencing geothermal energy mining, whose interaction and the relationship between the thermo-hydro-mechanical (THM) coupling determine the stability of reservoir rock mass. As shown in Table 2, we select three representative cases to discuss the effect of injection-production on the solid stress field, safety factor, and fracture permeability in the fractured reservoir. The injection pressure required to maintain normal injection-production for the selected three cases are 53.27–72.84 MPa, 57.57–98.19 MPa, and 63.84–123.66 MPa, respectively.

**Table 2:** Parameter Settings for the three representative cases selected.

Case #	Fracture aperture (mm)	Fracture length (m)	Fracture orientation	Injection rate (kg/s)
I	0.1–1.0	50–150	Random distribution	40
II	0.1–1.0			60
III	0.5			40

**Figure 13:**
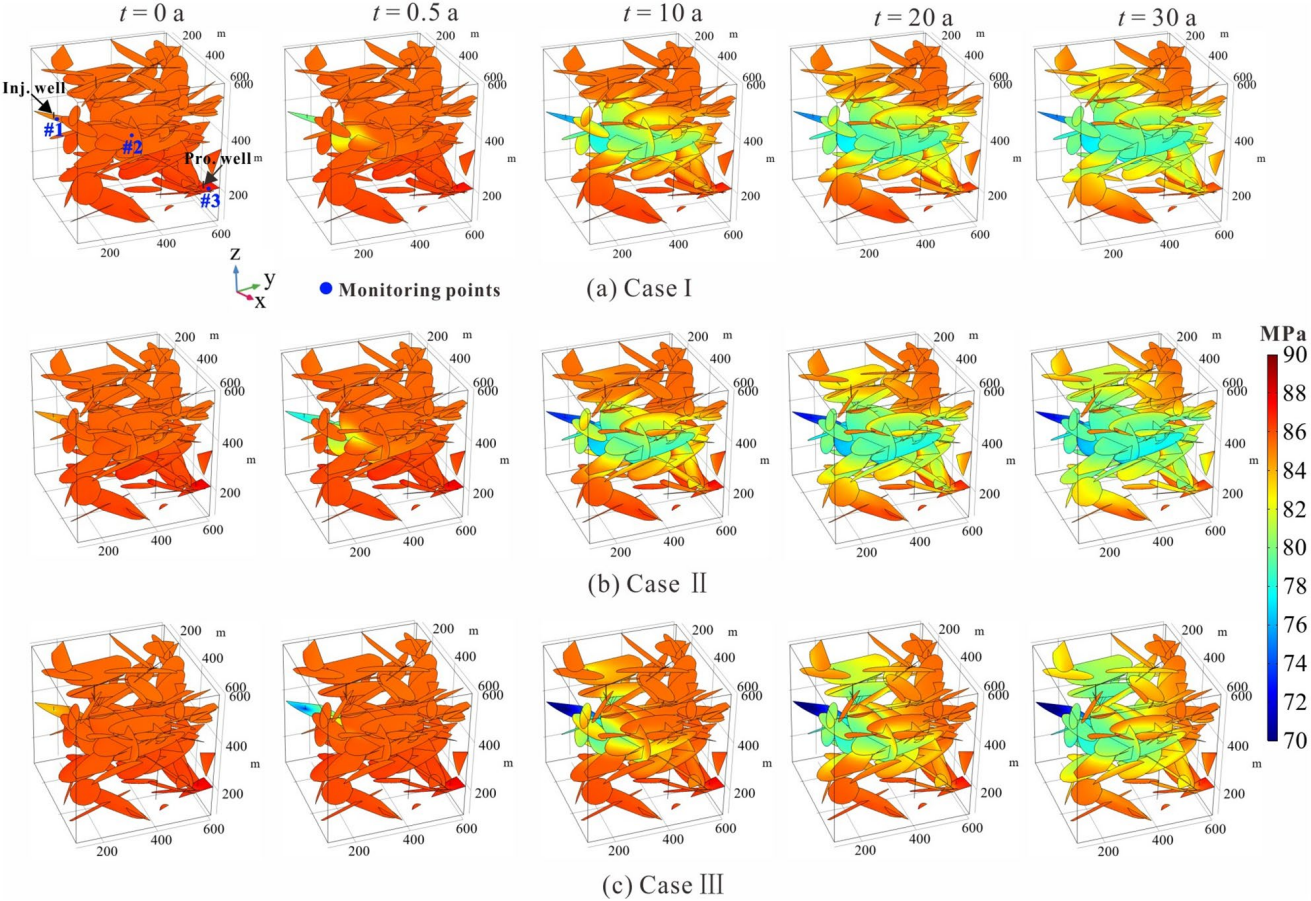
Evolution of the effective stress with time for three representative cases.

Figure 13 shows the spatial and temporal evolution of the effective stress in the fractured rock domain during the injection-production. The system is in its initial state (*t* = 0 a), the effective stress field exhibits a slightly heterogeneous pattern due to the presences of fracture network and wells. When low-temperature working fluid starts to be injected in the SRV (*t* = 0.5 a), the initial stress equilibrium is immediately broken. As the injection-production time proceeds, the stress field exhibits a strongly heterogeneous pattern along the main migration path of the working fluid. It can be observed that the most significant stress disturbance occurs in those fractures that are originally connected to the injection well, thereby the minimum effective stress occurs near the injection well, which is attributed to the poor-elastic effect. The stress disturbances of Cases II and III are stronger than those of Case I at any time, because their injection pressures are higher during operation.

The evolution of the solid stress field results in the change of fracture permeability and the stability of fracture wall rock. We have chosen three monitoring points to assess the variations in permeability and stability of fractures in the fractured EGS. As the fracture network remains the same, the positions of these three monitoring points are consistent across all three cases, as show in Fig. 13a. The chosen monitoring point #1 is located within the fracture that directly connect to the injection well, with the greatest change in effective stress. In addition, monitoring point #2 is selected at the center position of the fractured reservoir, while monitoring point #3 is chosen within the fracture that directly connect to the production well. As the distance from the injection well increases, the variation of the effective stress at the monitoring points weakens.

**Figure 14:**
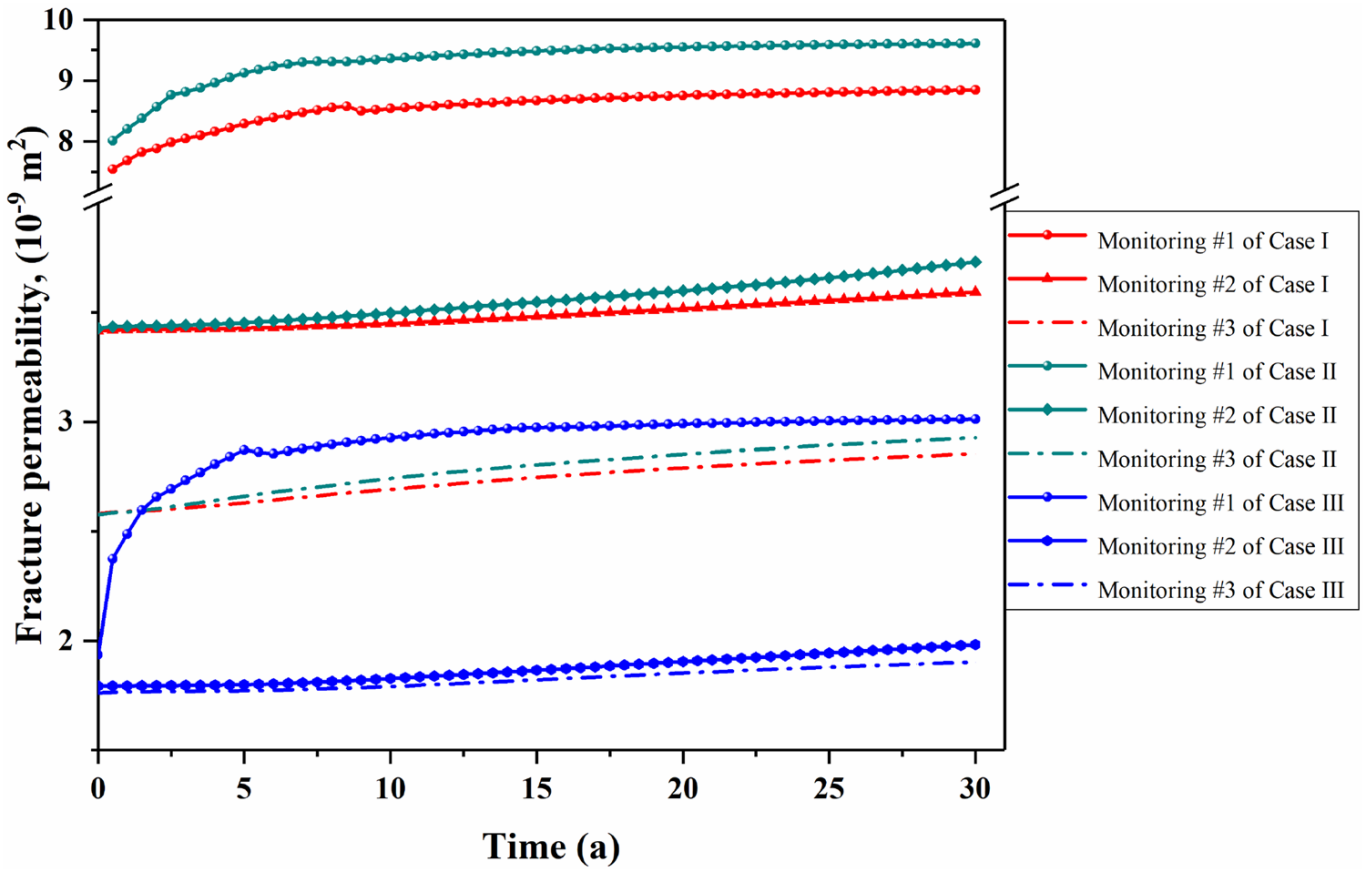
Permeability variation of the three monitoring points with time under THM coupling condition.

Herein, the fracture permeability is calculated by Eq. (15), which is governed by the fracture aperture and stress field. Thus, the variation of fracture permeability reflects the disturbance intensity of the stress field. Figure 14 presents the variation of fracture permeability with time at different monitoring points. In all three cases, the permeability of the fractures that directly connect to the injection well increases significantly within 5 years, then experiences a moderate increase until the end of operation, as suggested by the variation curves of the corresponding monitoring point #1. During the entire operation period, the permeability of the monitoring point #1 in the three cases increased by 34.7%, 43.9%, and 55.2%, respectively. Away from the injection well, the fractures do not witness much permeability variation response, as shown in the curves of the corresponding monitoring points #2 and #3.

**Figure 15:**
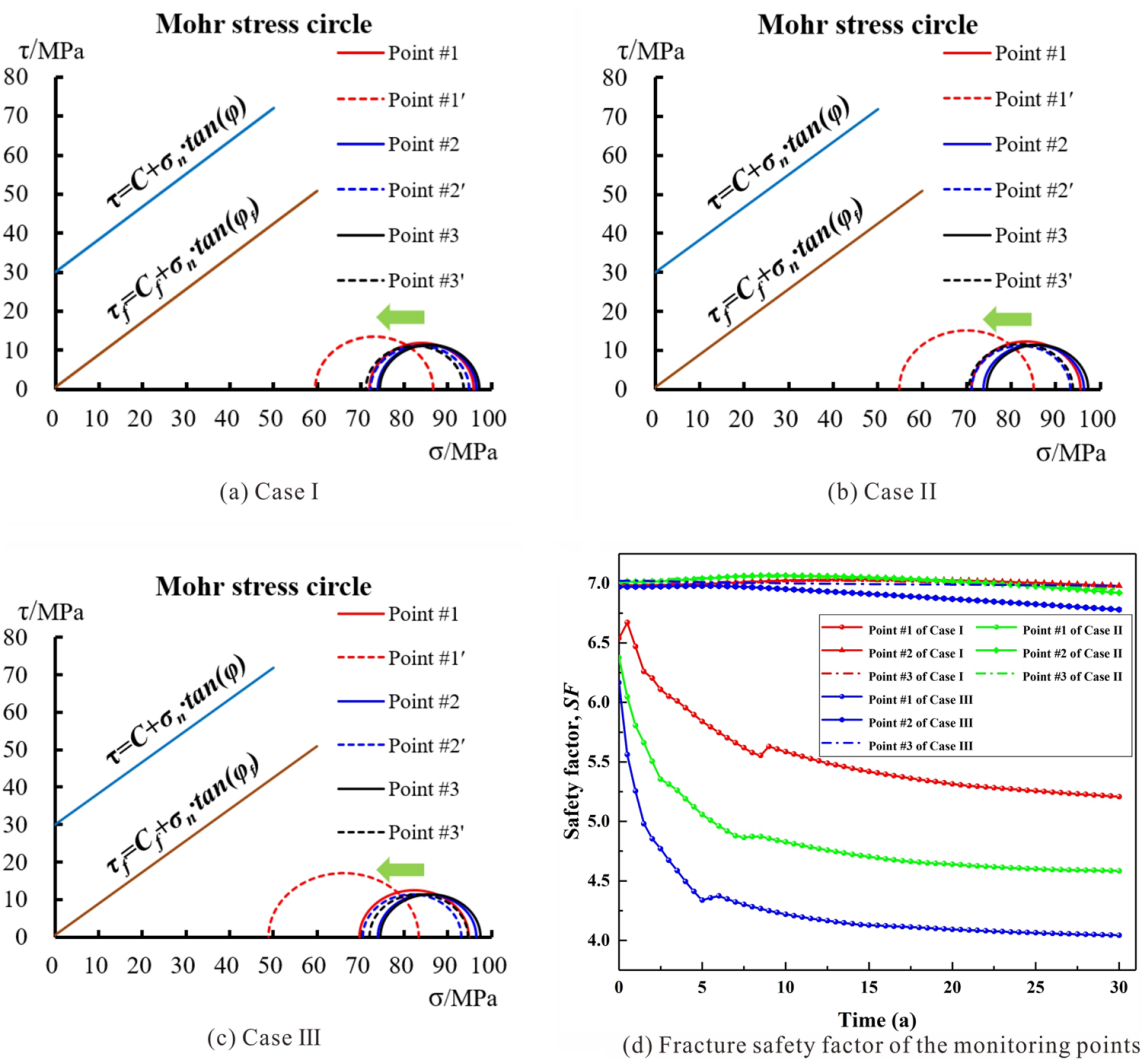
(**a**–**c**) The variation of Mohr stress circles at the monitoring point, in which *C* = 30 MPa, *φ* = 45°, *C*_*f*_ = 0.5 MPa, and *φ*_*f*_ = 40°, Point #1, #2 and #3 represent the initial stress states at the monitoring points, and Point #1′, #2′ and #3′ represent the stress states at the end of operation period; (**d**) Safety factor variation of the monitoring points with time under THM coupling condition.

The Mohr–Coulomb rock strength criterion is applied to assess the risk of failure of the SRV during the mining process. The Mohr circle, strength, and pressure are interrelated parameters that describe the geomechanical properties of a reservoir. Mohr circle is a plot of the stress state at a single point in a material, with the horizontal and vertical axes representing the normal and shear stresses acting on the material, which can help us predict the behaviors of reservoir rock during EGS operation. In this study, the cohesive strength (*C*) and internal friction angle (*φ*) of the granite were determined to be 30 MPa and 45°, respectively, while the cohesive strength (*C*_*f*_) and internal friction angle (*φ*_*f*_) of the fracture surface were determined to be 0.5 MPa and 40°, respectively. Based on this, the strength envelop of the fractured rock was determined and is shown in Fig. 15a–c. When the cold working fluid starts to be injected into EGS, the stress state variation in the reservoir instantly occurs to accommodate the transient fluid pressurization and thermally induced stress. As the mining time continues, the THM coupling behavior in EGS will lead to the continuous change in rock stress state, thus changing the size and position of the Mohr stress circle at the same monitoring point. For all three cases, we still choose the three representative monitoring points #1, #2 and #3 to evaluate the stability of the reservoir rock mass; at the end of the operation, the Mohr stress circles for the corresponding monitoring points are #1′, #2′ and #3′. As shown in Fig. 15a–c, it can be observed that the stress state of monitoring point #1 adjacent to the injection well undergoes the greatest variation throughout the entire operation period under the same conditions. In comparison with Case I, monitoring point #1 in Case III with a higher injection pressure exhibits the most significant changes in stress state, including leftward shift of the stress circle position and a significant increase in the circle radius. However, the Mohr circles for different simulation cases are all located below the strength envelope, indicating that no shear failure occurred at any monitoring points.

In addition, Fig. 15d shows the variation of safety factor over time at the corresponding monitoring point. In all three cases, the safety factor of the fracture that directly connect to the injection well drops significantly within 5 years, then experiences a moderate decline until the end of operation, as suggested by the variation curves of the corresponding monitoring point #1 (Fig. 15b). Away from the injection well, the variation of the effective stress in the fractured rocks weakens, thus, the safety factor at monitoring points #2 and #3 in all three cases not much response appears. In a 30-year operation time, the safety factors at monitoring point #1 in the three selected cases decreased by 20.3%, 28.2%, and 34.5%, respectively. The safety factor at monitoring point #1 decreases in Case III is more pronounced than in the other two cases. The safety factor curve verifies the analysis results of the Mohr stress circle, thus indicating that under the background conditions of the HDR site in the Gonghe Basin, HDR mining operation employing an injection rate of 40–60 kg/s in the fracture network with an aperture of 0.1–1 mm will not cause shear failure to the reservoir rock.

To sum up, we found that the Case III has a more remarkable response to the variations of the effective stress, fracture permeability, and safety factor, this is because Case III requires a higher injection pressure to maintain injection-production operation. Compared to Cases 1 and 2, the high injection pressure is evidently caused by the small average fracture aperture. Therefore, an appropriate fracture aperture is key to maintaining the efficient operation of EGS. As demonstrated in "Fracture aperture" section, when developing EGS in a deep-seated geological environment, the optimal fracture aperture should be at least kept in a range of 0.5–1.0 mm in a self-propping fracture system. For spatial differences in a specific case, the more remarkable response of the mentioned three parameters occurs at the position near the injection well, which is attributed to the high pressure and notable temperature drop. Thus, we also should strictly limit the injection pressure and ensure that the fractured rock near the injection well will not be destabilized.

## Conclusion

Modeling discrete fracture networks (DFN) helps to understand the characteristics of fluid flow, heat transfer, and mechanics in fractured rocks and reservoirs. In this work, DFN was used for the investigation of energy extraction from an enhanced geothermal system (EGS). The effects of fracture network morphology, fracture dip, fracture aperture, fracture length, and injection parameters on the production performance of EGS are studied. The conclusions:

1. In a fractured reservoir, both fracture and matrix have important roles for heat exchange, but the interconnected fractures between injection and production wells determine the flow path of the working fluid. When the working fluid is injected into the fractured rock, high fluid pressure first builds up along the interconnected fractures close to the injection well, resulting in a strong disturbance zone of temperature and pressure fields. Over time, the temperature, pressure, and stress perturbation zones diffuse asynchronously in different directions, in which the stress field perturbation always lags behind the other two.
2. For a certain number of fractures, the fracture network geometry slightly affects the injection pressure but significantly affects the heat production. The influence of fracture geometry on the production performance is mainly related to the fracture dip. The fracture dip apparently influences the production temperature and output thermal power, with the increase of fracture dip, the production temperature and thermal output power decrease significantly over 30 years of operation.
3. In a self-propping fracture system, the fracture aperture highly determines the fluid flow behavior. A fractured reservoir with smaller fracture aperture shows the worse fluid injection performance but the better geothermal extraction performance. When the average aperture of the fracture network is less than 0.5 mm, the required injection pressure far exceeds the minimum principal stress of the reservoir, posing a threat to reservoir safety. However, when the average aperture of the fracture network is larger than 1.0 mm, the injection pressure required is smaller, but there is a significant decrease in production temperature and output thermal power in a short period of time, which does not meet the economic and efficient EGS concept. Thus, the optimal fracture aperture should be kept in a range of 0.5–1.0 mm in a self-propping fracture system.
4. The effect of fracture length on EGS performance highly depends on the fracture orientation. Excessive increase of fracture length towards injection-production well is detrimental to heat extraction, but the increase of fracture length perpendicular to the direction of injection-production well can improve the thermal extraction performance.
5. For a fractured geothermal reservoir, the effect of injection mass flow rate on EGS performance is significantly greater than that of injection temperature. Increasing the injection flow rate can significantly improve the output thermal power, but will lead to a higher injection pressure and lower production temperature. In addition, the geothermal extraction ratio increases with the increase of injection flow rate, but decreases with the increase of injection temperature. Thus, to achieve a cost-effective successful EGS, it is feasible to inject water at the right temperature at a moderate flow rate.
6. The scenario with a higher injection pressure has a more remarkable response to the variations of the effective stress, fracture permeability, and safety factor. During the injection-production process, the most significant stress disturbance occurs in those fractures that are originally connected to the injection well, where high fluid pressure first builds up. Then, the strong stress field disturbance leads to significant variations in these fractures, including increased permeability and decreased safety factor.

## Data Availability

The datasets used and/or analysed during the current study available from the corresponding author on reasonable request.

## List of symbols

$\rho_{w}$: Working fluid density, kg/m^3^
$C_{w}$: Specific heat capacity of working fluid, J/(kg K)
$\lambda_{w}$: Thermal conductivity of working fluid, W/(m K)
$k$: Rock matrix permeability, m^2^
$$\phi_{{\text{f}}}$$: Fracture porosity
$C_{s}$: Specific heat capacity of rock, J/(kg K)
$\lambda_{s}$: Thermal conductivity of rock, W/(m K)
$\nu$: Poisson’s ratio of the reservoir rock
$\alpha_{T}$: Thermal expansion coefficient of reservoir rock, 1/K
$k_{f0}$: Initial fracture permeability, m^2^
$P_{inj}$: Injection pressure, MPa
$W_{h}$: Output thermal power, MW
$T_{inj}$: Injection fluid temperature, ℃
$\mu$: Working fluid viscosity, Pa s
$\phi$: Reservoir rock porosity
${\mathbf{U}}$: Fluid flow velocity, m/s
$d_{f}$: Fracture aperture, mm
$\rho_{s}$: Reservoir rock density, kg/m^3^
$E$: Elastic modulus of rock, GPa
$$\alpha$$: Biot coefficient
$$SF$$: Safety factor
$$k_{f}$$: Fracture permeability, m^2^
$$T_{pro}$$: Production temperature, ℃
*ξ*: Heat extraction ratio
$Q_{inj}$: Injection fluid mass rate, kg/s
